# Identification of dopamine receptors across the extant avian family tree and analysis with other clades uncovers a polyploid expansion among vertebrates

**DOI:** 10.3389/fnins.2015.00361

**Published:** 2015-10-07

**Authors:** Asher Haug-Baltzell, Erich D. Jarvis, Fiona M. McCarthy, Eric Lyons

**Affiliations:** ^1^Arizona Biological/Biomedical Sciences Program, University of ArizonaTucson, AZ, USA; ^2^Genetics GIDP, University of ArizonaTucson, AZ, USA; ^3^Department of Neurobiology, Duke University Medical CenterDurham, NC, USA; ^4^Howard Hughes Medical InstituteChevy Chase, MD, USA; ^5^School of Animal and Comparative Biomedical Sciences, University of ArizonaTucson, AZ, USA; ^6^BIO5 Institute, University of ArizonaTucson, AZ, USA; ^7^The School of Plant Sciences, University of ArizonaTucson, AZ, USA

**Keywords:** dopamine receptors, comparative genomics, genome evolution, polyploidy, whole genome duplication

## Abstract

Dopamine is an important central nervous system transmitter that functions through two classes of receptors (D1 and D2) to influence a diverse range of biological processes in vertebrates. With roles in regulating neural activity, behavior, and gene expression, there has been great interest in understanding the function and evolution dopamine and its receptors. In this study, we use a combination of sequence analyses, microsynteny analyses, and phylogenetic relationships to identify and characterize both the D1 (DRD1A, DRD1B, DRD1C, and DRD1E) and D2 (DRD2, DRD3, and DRD4) dopamine receptor gene families in 43 recently sequenced bird genomes representing the major ordinal lineages across the avian family tree. We show that the common ancestor of all birds possessed at least seven D1 and D2 receptors, followed by subsequent independent losses in some lineages of modern birds. Through comparisons with other vertebrate and invertebrate species we show that two of the D1 receptors, DRD1A and DRD1B, and two of the D2 receptors, DRD2 and DRD3, originated from a whole genome duplication event early in the vertebrate lineage, providing the first conclusive evidence of the origin of these highly conserved receptors. Our findings provide insight into the evolutionary development of an important modulatory component of the central nervous system in vertebrates, and will help further unravel the complex evolutionary and functional relationships among dopamine receptors.

## Introduction

Dopamine (DA) is an important neurotransmitter that functions in the central nervous system of vertebrates. DA functions through two main classes of receptors, designated D1 (Class 1) and D2 (Class 2), to influence neural activity, behavior, and gene expression (Stoof and Kebabian, 1984; Beaulieu and Gainetdinov, 2011). All DA receptors are integral membrane proteins and belong to the rhodopsin family of G protein-coupled receptors, but the two classes are structurally, functionally, and genetically distinct. D1 receptors consist of a single exon open reading frame with no introns, contain a short third cytoplasmic loop and long cytoplasmic C-terminal stretch, and activate adenylyl cyclase resulting in increased cAMP levels (Callier et al., 2003; Le Crom et al., 2003; Yamamoto et al., 2013). Conversely, the D2 receptors possess multiple exons and introns, have a long third cytoplasmic loop and a short C-terminus stretch that remains anchored in the membrane, and reduced adenysyl cyclase activity (Callier et al., 2003; Le Crom et al., 2003). The source of DA and the expression of its associated receptors are well conserved between birds and mammals, and are important in brain modulation of many behaviors, including reproduction, learning, vocalization, addiction, and reward, making them of particular interest in both health and agricultural applications (Schnell et al., 1999; Chaiseha et al., 2003; Sasaki et al., 2006; Sartsoongnoen et al., 2008; Kubikova et al., 2010).

Dopamine and its receptors have been studied both functionally and evolutionarily. The D1 family is currently known to include four paralogous receptors DRD1A (human DRD1), DRD1B/X (human DRD5), DRD1C/D, and DRD1E (Callier et al., 2003; Yamamoto et al., 2013). The D2 family includes three paralogous receptors DRD2, DRD3, and DRD4 (Callier et al., 2003). Not all dopamine receptors are present in all species, and lineage-specific duplications have increased receptor number in some species (Yamamoto and Vernier, 2011). For example, only two D1 receptors DRD1A and DRD1B have been identified in mammals, while in birds a combination of DRD1A, DRD1B, DRD1C, and/or DRD1E may be present (Kubikova et al., 2010; Yamamoto et al., 2013). In the teleost fishes, DRD1B has duplicated and formed the DRD1X paralog (Yamamoto et al., 2013). It is commonly accepted that the two classes of vertebrate dopamine receptors are no more closely related to each other than to other classes of monoamine receptors, suggesting that they likely converged upon binding dopamine (Callier et al., 2003; Yamamoto and Vernier, 2011; Robertson et al., 2012; Yamamoto et al., 2013). However, questions still remain regarding the complex functional and evolutionary relationships between the receptors of each class.

Here, we identified and characterized D1 (DRD1A, DRD1B, DRD1C, and DRD1E) and D2 (DRD2, DRD3, and DRD4) dopamine receptor gene families in 43 recently sequenced bird genomes spanning the avian family tree at the ordinal level (Table 1) (Zhang et al., 2014). We used phylogenetic relationships, macro- and microsynteny, and comparisons to homologous receptors in other vertebrate genomes to make significant advances in our understanding of the evolutionary origins of D1 and D2 receptors among birds and other vertebrates.

**Table 1:** **Avian genome information and summary of gene absence confirmation by microsynteny and BLAST searches of raw sequencing reads**.

**Species**	**Common name**	**NCBI bioproject accession**	**Missing gene**	**Microsynteny**	**BLAST coverage**	**Citation**
*T. guttata*	Zebra Finch	PRJNA17289	DRD1E	Unidentified	N/A	Warren et al., 2010
*G. fortis*	Medium Ground Finch	PRJNA156703	DRD4	Unidentified	Good	Zhang et al., 2014
*C. brachyrhynchos*	American Crow	PRJNA212869	N/A	N/A	N/A	Zhang et al., 2014
*M. vitellinus*	Golden Collared Manakin	PRJNA212872	DRD1C	Absent	Poor (30%)	Zhang et al., 2014
			DRD1E	Absent	Good	
*A. chloris*	Rifleman	PRJNA212877	DRD4	Unidentified	Good	Zhang et al., 2014
*M. undulatus*	Budgerigar	PRJEB1588	DRD1E	Absent	Poor (34%)	Ganapathy et al., 2014
*N. notabilis*	Kea	PRJNA212900	N/A	N/A	N/A	Zhang et al., 2014
*F. peregrinus*	Peregrine Falcon	PRJNA159791	N/A	N/A	N/A	Zhan et al., 2013
*C. cristata*	Red Legged Seriema	PRJNA212889	DRD4	Unidentified	Good	Zhang et al., 2014
*M. numbicus*	Northern Carmine Bee Eater	PRJNA212898	DRD4	Unidentified	Good	Zhang et al., 2014
*P. pubescens*	Downy Woodpecker	PRJNA212874	N/A	N/A	N/A	Zhang et al., 2014
*B. silvestris*	Javan Rhinoceros Hornbill	PRJNA212887	DRD4	Unidentified	Good	Zhang et al., 2014
*A. vittatum*	Bar Tailed Trogon	PRJNA212878	DRD4	Unidentified	Good	Zhang et al., 2014
*L. discolor*	Cuckoo Roller	PRJNA212897	N/A	N/A	N/A	Zhang et al., 2014
*C. striatus*	Speckled Mousebird	PRJNA212892	DRD1E	Unidentified	Good	Zhang et al., 2014
			DRD4	Unidentified	Good	
*T. alba*	Barn Owl	PRJNA212909	DRD4	Unidentified	Good	Zhang et al., 2014
*H. albicilla*	White Tailed Eagle	PRJNA212896	DRD1B	Unidentified	Good	Zhang et al., 2014
*P. crispus*	Dalmatian Pelican	PRJNA212901	DRD4	Unidentified	Good	Zhang et al., 2014
*N. nippon*	Crested Ibis	PRJNA232572	N/A	N/A	N/A	Zhang et al., 2014
*P. carbo*	Creat Black Cormorant	PRJNA212903	DRD1C	Absent	Poor (34%)	Zhang et al., 2014
			DRD4	Unidentified	Good	
*F. glacialis*	Northern Fulmar	PRJNA212894	N/A	N/A	N/A	Zhang et al., 2014
*P. adeliae*	Adelie Penguin	PRJNA235983	N/A	N/A	Good	Zhang et al., 2014
*G. stellata*	Red Throated Loon	PRJNA212895	DRD4	Unidentified	Good	Zhang et al., 2014
*P. lepturus*	White Tailed Tropicbird	PRJNA212902	DRD4	Unidentified	Good	Zhang et al., 2014
*E. helias*	Sunbittern	PRJNA212893	DRD4	Unidentified	Good	Zhang et al., 2014
*C. vociferous*	Killdeer	PRJNA212867	DRD4	Unidentified	Good	Zhang et al., 2014
*O. hoazin*	Hoatzin	PRJNA212873	DRD4	Unidentified	Good	Zhang et al., 2014
*C. anna*	Anna's Hummingbird	PRJNA212866	DRD1C	Absent	Poor (15%)	Zhang et al., 2014
			DRD1E	Unidentified	Poor (35%)	
			DRD4	Unidentified	Good	
*C. pelagica*	Chimney Swift	PRJNA210808	DRD4	Unidentified	Good	Zhang et al., 2014
*A. carolinensis*	Night Jar	PRJNA212888	N/A	N/A	N/A	Zhang et al., 2014
*C. macqueenii*	Macqueen Bustard	PRJNA212891	DRD4	Unidentified	Good	Zhang et al., 2014
*T. erythrolophus*	Angola Turaco	PRJNA212908	DRD4	Unidentified	Good	Zhang et al., 2014
*M. unicolor*	Brown Mesite	PRJNA212899	DRD4	Unidentified	Good	Zhang et al., 2014
*P. guturalis*	Yellow Throated Sandgrouse	PRJNA212906	DRD4	Unidentified	Good	Zhang et al., 2014
*C. livia*	Domestic Pigeon	PRJNA167554	DRD4	Unidentified	Poor (33%)	Shapiro et al., 2013
*P. ruber*	Caribbean Flamingo	PRJNA212904	N/A	N/A	N/A	Zhang et al., 2014
*P. cristatus*	Great Crested Grebe	PRJNA212905	DRD4	Unidentified	Good	Zhang et al., 2014
*G. gallus*	Chicken	PRJNA13342	DRD1E	Unidentified	Good	Hillier et al., 2004
*M. gallopavo*	Turkey	PRJNA42129	DRD4	Unidentified	N/A	Dalloul et al., 2010
*A. domestica*	Peking Duck	PRJNA46621	DRD4	Unidentified	Good	Huang et al., 2013
*T. guttatus*	White Throated Tinamou	PRJNA212876	DRD1E	Unidentified	Good	Zhang et al., 2014
			DRD4	Unidentified	Good	
*S. camelus*	Ostrich	PRJNA212875	DRD4	Unidentified	Good	Zhang et al., 2014

*All bird species, including both scientific and common names, investigated in this study. Table is organized in order of bird phylogeny (**Figure 2**). Each species includes a reference to the original sequencing publication, the NCBI BioProject accession number for the raw data, and a summary of evidence supporting absence or lack of identification for each unidentified receptor. For microsynteny analyses, “Absent” refers to gene appearing deleted in microsynteny analyses, while “Unidentified” indicates sequence similarity but no intact gene annotation. BLAST coverage summarizes the extent to which raw reads covered a query receptor sequence when raw sequencing data (obtained from NCBI) was BLAST searched. “Poor” coverage (≤ 35%) suggests receptor is absent, while “good” (>35%) coverage suggests receptor may be present. In cases where a receptor is indicated as missing, but raw reads coverage is listed as “N/A,” raw reads for that species were either unavailable or corrupted and thus the analysis could not be performed on that species (i.e., Zebra Finch). Raw results and additional explanation are compiled in Supplementary File 1*.

## Materials and methods

### Sequence data

To identify putative dopamine receptors across the avian lineage, we used the DNA and protein coding sequences of previously identified D1 and D2 receptors in Chicken, Turkey, and Zebra Finch to BLAST against 43 unmasked avian genome sequences from the Avian Phylogenomics Consortium (Jarvis et al., 2014; Zhang et al., 2014). The 43 species were chosen based on being representative of all major orders of modern birds, and having a now well-vetted species-level phylogenetic trees (Jarvis et al., 2014). All avian BLAST query reference sequences were obtained from the Ensembl Database v81 (Flicek et al., 2014). Accession numbers are listed in **Table 3**. BLAST searches were performed with CoGeBlast (Lyons and Freeling, 2008), and results were filtered based on *e*-value and HSP coverage to remove false positives (Schnable and Lyons, 2012). The remaining putative DA receptor gene protein coding sequences were exported and compiled into multi-FASTA files. To serve as an outgroup, the protein coding sequence for a Ciona D1-like receptor was acquired from GenScript (GenScript)^1^, and appended to the multi-FASTA (Supplementary File 4).

To infer an evolutionary model for the origin of DA receptors, DA receptor protein coding sequences were obtained for a range of vertebrate and invertebrate species, including human, mouse, chicken, turkey, peregrine falcon, alligator, fugu, lamprey, ciona, and drosophila. Protein coding sequences for chicken, turkey, human, and mouse D1 and D2 receptors, and fugu D2 receptors were obtained from Ensembl. Fugu D1 sequences were identified with CoGeBLAST using the orthologous chicken gene coding sequences as queries and confirmed via microsynteny analyses (Supplemental File 3). The remaining bird sequences were identified in this study. Alligator sequences were obtained using CoGe tool SynFind (https://genomevolution.org/CoGe/SynFind.pl) using bird receptors as queries to identify orthologous genes (and genomic regions) in alligator. Ciona D1-like was obtained from GenScript. To serve as the outgroup, Drosophila dopamine 1 receptor (Dop1) protein coding sequence was acquired from Flybase (Pierre et al., 2014). All coding sequences were compiled into a multi-FASTA file (Supplementary File 5) for alignment and phylogenetic analysis. Accession IDs for all sequences obtained from external resources other than CoGe may be found in **Table 3**.

### Phylogenetic analysis

**Figure 1:**
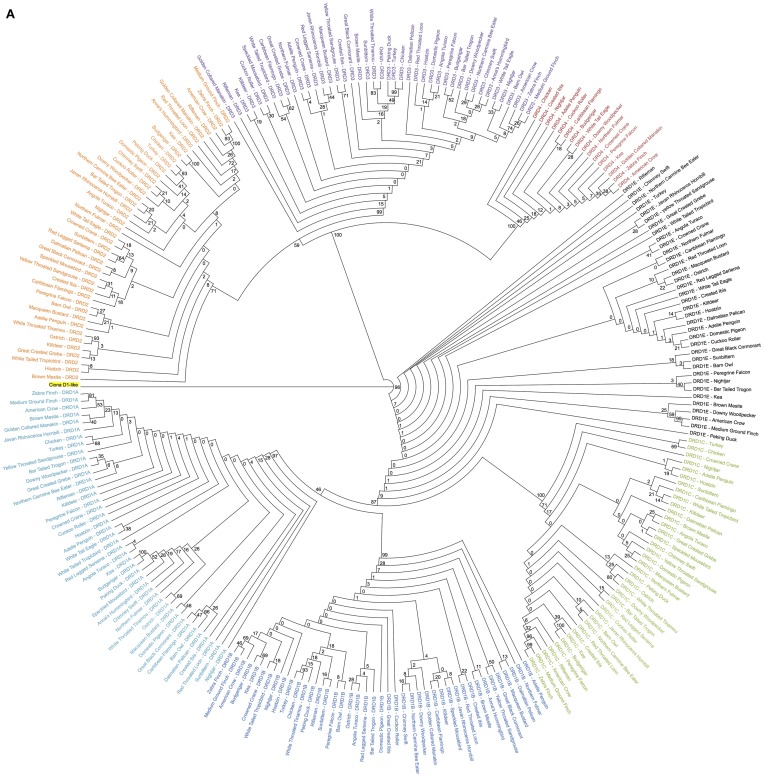

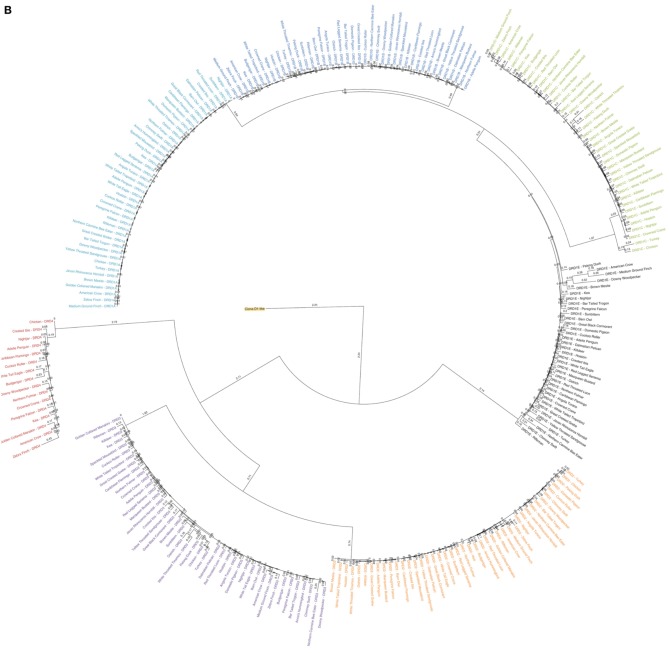
**Phylogenetic analysis of dopamine receptors in 43 bird species**. Inferred maximum-likelihood evolutionary phylogeny of the 7 DA receptors found in birds based on protein sequences. The phylogeny is composed of two major clades: (1) The Class 1 (D1) receptors, with DRD1A in light blue, DRD1B in dark blue, DRD1C in green and DRD1E in black; and (2) The Class 2 (D2) receptors, with DRD2 in orange, DRD3 in purple, and DRD4 in red. Trees are rooted on the Ciona D1-like receptor (highlighted yellow). Calculations were performed using RAxML 8.1.2 (Stamatakis, 2014). **(A)** Cladogram of phylogenetic relationships with support values based on 100 bootstrap replicates. **(B)** Phylogeny of receptor relationships with branch lengths indicated to show rates of divergence.

All phylogenetic analyses were generated from protein-coding sequences (Figures 1A,B, **7A**). For each analysis, the respective multi-FASTA file(s) were uploaded to the iPlant Data Store (Goff et al., 2011). Sequences were aligned into PHYLIP Interleaved format using the iPlant Discovery Environment (DE) implementation of MUSCLE 3.8.31 (Supplementary Files 6, 7) (Edgar, 2004). To confirm the validity of programmatic (fully automated) alignment, the multiple sequence alignments used for construction of all phylogenies were visualized using Jalview (Waterhouse et al., 2009). Minor errors were observed, but manual adjustment did not create well-supported (bootstrap values) improvements to the gene-tree in regards to species-level relationships (Supplemental File 2: Figures S2A, S2B). As such, automatic alignments were used for all analyses, which also increases the reproducibility of our study. Phylogenetic analyses were performed on the aligned sequences using a customized iPlant implementation of RAxML 8.1.2 2 (“Protein-Based RAxML Analysis”) (Goff et al., 2011; Stamatakis, 2014). Best ML trees were calculated from 20 replicates using the PROTGAMMA Model and WAG AA substitution matrix. To generate a final tree with bootstrap support values, 100 bootstrap replicates were computed, using the same model as for best tree calculations, and the values were drawn onto the best tree using the PROTCAT model. Phylogenetic analyses can be regenerated from the Supplementary Material multi-FASTA files (Supplementary Files 4, 5) with the iPlant DE tools “MUSCLE 3.8.31” and “Protein-Based RAxML Analysis.” Alternately, phylogenies can be regenerated directly from the multiple sequence alignments (Supplementary Files 6, 7) using only iPlant DE tool “Protein-Based RAxML Analysis” or any RAxML 8.1.2 installation. Tree visualizations with bootstrap support values were performed using Dendroscope (Huson and Scornavacca, 2012), and visualizations with branch lengths using FigTree (http://tree.bio.ed.ac.uk/software/figtree/).

### Synteny analysis

#### Microsynteny

Genomic microsynteny analyses were performed to confirm putative gene identity and to differentiate between absent and unannotated receptors. All microsynteny visualizations were generated using the GEvo tool, available through the comparative genomics platform CoGe (www.genomevolution.org). GEvo plots annotated gene features along chromosome panels of user-designated lengths, and uses LAST algorithms to identify regions of high sequence similarity. Regions with high sequence similarity are physically connected using colored boxes and lines. Annotated features overlapped by these regions are colored purple, so that users can infer collinear gene order of homologous gene pairs between multiple species/chromosomes. All GEvo microsynteny analyses have links provided in figure legends to regenerate the analysis and visualization.

#### Genome wide synteny

To identify genome wide duplication patterns and investigate the nature of the DA receptor duplications, the CoGe tool SynMap was used to generate a full-genome syntenic dot plot between Chicken and Turkey (**Figure 5**). Synonymous mutation (Ks) values for each syntenic gene pair were calculated using CODEML (Yang, 2007). Ks values were plotted onto a histogram, and a color scheme was applied across the histogram. The corresponding colors were mapped onto each dot to visualize genome-wide duplication event patterning. Dopamine receptor genes were located on the plot to determine how their duplication history fits with the genome-wide duplication history. The SynMap analysis has a link provided in the figure legend to regenerate the analysis and visualization.

### Raw reads BLAST search

To confirm the gene absences indicated by microsynteny analysis, BLAST searches were performed against the raw sequencing reads for target bird genomes. Raw reads were extracted from SRA files obtained from the NCBI BioProject for each avian species (Table 1), and BLAST was performed using the NCBI blastn suite (Altschul et al., 1990). Program selection was optimized for highly similar sequences (megablast), with the following modifications made to default settings: “Expect threshold = 5,” “Word size = 16,” “Gap Costs = Existence: 5, Extension: 2.” For each search, the query sequence was composed of 5–6 known coding sequences for the receptor of interest. For each species investigated, a BLAST for DRD1A (always present) served as a control. All raw read BLAST search results are included in Supplemental File 1, and both BLAST and microsynteny results are summarized in Table 1.

### Human ortholog comparisons

To test for possible additional evidence into the WGD origin of the DRD1A/DRD1B receptors, maps comparing human chromosome 4 (containing DRD1, the DRD1A ortholog) and chromosome 5 (containing DRD5, the DRD1B ortholog) were generated using “Paralogons in the Human Genome” tool from the Wolfe lab (http://wolfe.ucd.ie/cgi-bin/dup_528/chrom_plot) (McLysaght et al., 2002) (**Figure 6A**). Minimum threshold for paired proteins per block was set as 12, as values greater than 6 were shown as statistically likely as having originated from a whole genome duplication event (McLysaght et al., 2002), and a threshold of 12 produced only a single block overlapping the region of interest in our analysis. For DRD2/DRD3 receptors, similar maps comparing human chromosome 11 (containing DRD2) and chromosome 3 (containing DRD3) were generated using a minimum threshold for paired proteins per block of 7 (**Figure 6B**). Specific locations of the receptor genes were marked on the map by comparison with the human reference genome. Similar maps were generated for the other DA receptor pairs, but genes did not fall directly within regions shown as duplicated (not shown).

## Results

### Identification and classification of DA receptors in bird species

Sequence comparisons (BLAST) were used to identify putative DA receptor encoding genes in the 43 sequenced bird genomes (Table 1) (Zhang et al., 2014). Maximum-likelihood phylogenies, rooted on the outgroup D1-like receptor from Ciona (a tunicate invertebrate), were constructed from protein coding sequences of the putative DA receptors using RAxML (Figure 1) (Stamatakis, 2014). Gene identity was inferred by presence within specific receptor clades and further confirmed by syntenic analysis. Putative genes that did not have both phylogenetic and syntenic evidence were discarded.

Overall topography of the receptor phylogeny was similar to previous reports, with two major monophyletic clades comprised of the two major classes of DA receptors, D1 and D2 (Figure 1). The D1 clade was broken into 4 major sub-clades, each containing exclusively DRD1A, DRD1B, DRD1C, or DRD1E receptors. Phylogenetic relationships between the receptors, (DRD1E,(DRD1C,(DRD1A,DRD1B))), were congruent with those reported by Yamamoto et al. (2013). Within the D1 class, DRD1E was the fastest diverging DA receptor family, followed by DRD1C, with DRD1A and DRD1B diverging slowest. Additionally, within individual receptor family clades, DRD1C and DRD1E diverged at a rate 2–4 times faster than DRD1A and DRD1C (Figure 1B). Receptor clades for DRD1A, DRD1B, and DRD1C clades were monophyletic while DRD1E was polyphyletic, supporting the findings of Yamamoto et al. (2013). However, our phylogeny had significantly higher bootstrap support for the nodes supporting these clades: 87 vs. 21 for the node separating DRD1C and DRD1A/1B; 46 vs. 17 for the node separating DRD1A and DRD1B (Figure 1A) (Yamamoto et al., 2013).

The D2 clade was broken into three major sub-clades, each monophyletic for DRD2, DRD3, and DRD4 receptors. Branching relationships were congruent with previously published relationships (DRD4,(DRD2,DRD3)), with DRD4 diverging fastest, both as a family within the D2 class and as individual receptors within the DRD4 clade (Le Crom et al., 2003). While we had similarly high bootstrap support (100 vs. 99) for the node separating DRD4 and DRD2/3, our bootstrap value for the node separating DRD2 and DRD3 was lower (59 vs. 100) (Figure 1A) (Le Crom et al., 2003).

Species relationships within each receptor clade were not congruent with the avian species tree phylogeny (Jarvis et al., 2014) except for closely related species on the terminal branches. This result was expected as recent findings show that: (1) gene trees almost never correlate perfectly with species trees; (2) single genes have lower resolution (bootstrap support) than thousands of genes or whole genomes; and (3) protein coding genes have lower phylogenetic signal due to high sequence conservation, including G-coupled protein receptors (Jarvis et al., 2014). Additionally, minor errors in the alignment resulting from the automated alignment did not appear to be contributing to these differences, as they were not remedied by manual corrections of the alignment (Supplemental File 2). Despite the discrepancies in species level relationships, gene-level relationships demonstrated high support and were congruent with those previously reported (Kubikova et al., 2010; Yamamoto et al., 2013).

### Independent preferential loss of derived DA receptors among bird species

**Figure 2:**
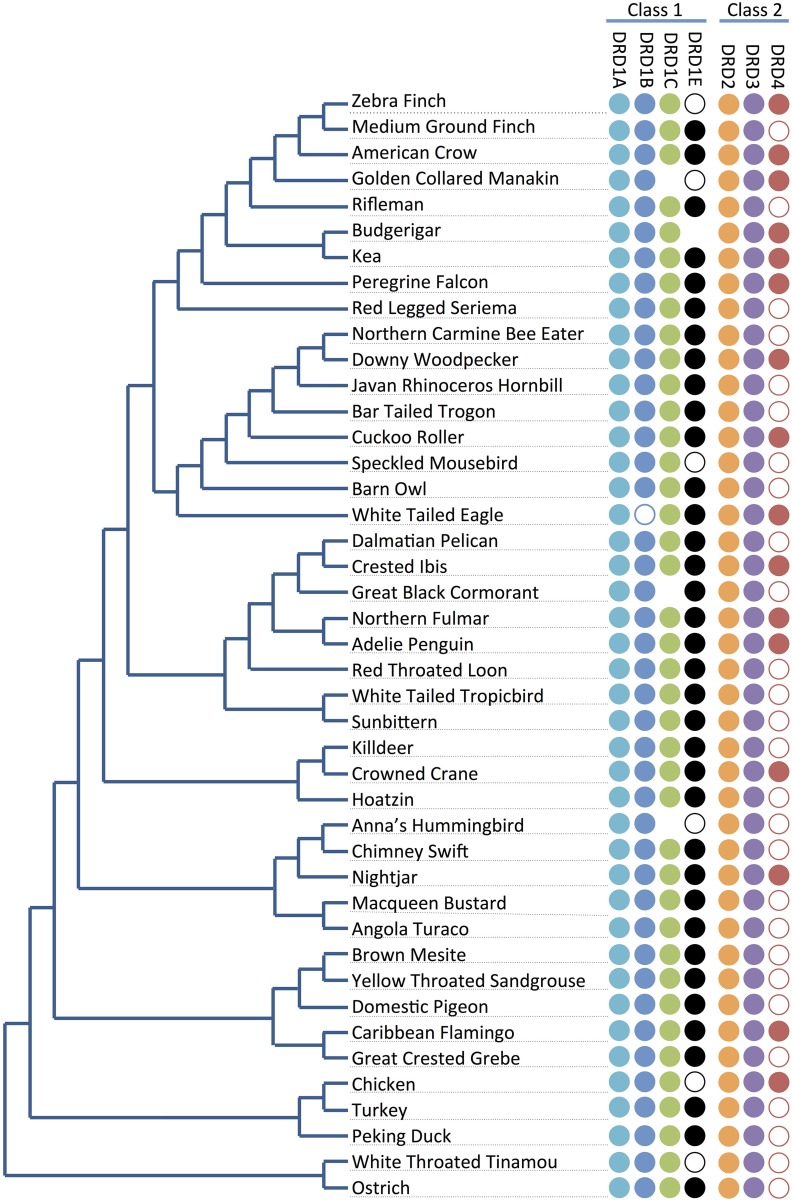
**Receptor presence/absence by species**. Presence and absence of dopamine receptors (right) across the phylogeny of the 43 investigated bird species (left; tree adapted from Jarvis et al., 2014). Full circles indicate receptor genes are present and identified. Empty circles indicate receptor genes not annotated in the genome, but with at least significant portions of the sequences identified by microsynteny analysis (e.g., Figure 3B) or BLAST searches against raw sequencing reads (Supplementary File 1). No circles represent receptor genes are likely absent in that species due to not being found through any analyses. Losses do not follow a phylogenetic pattern, and thus appear to be lost independently in different lineages.

**Figure 3:**
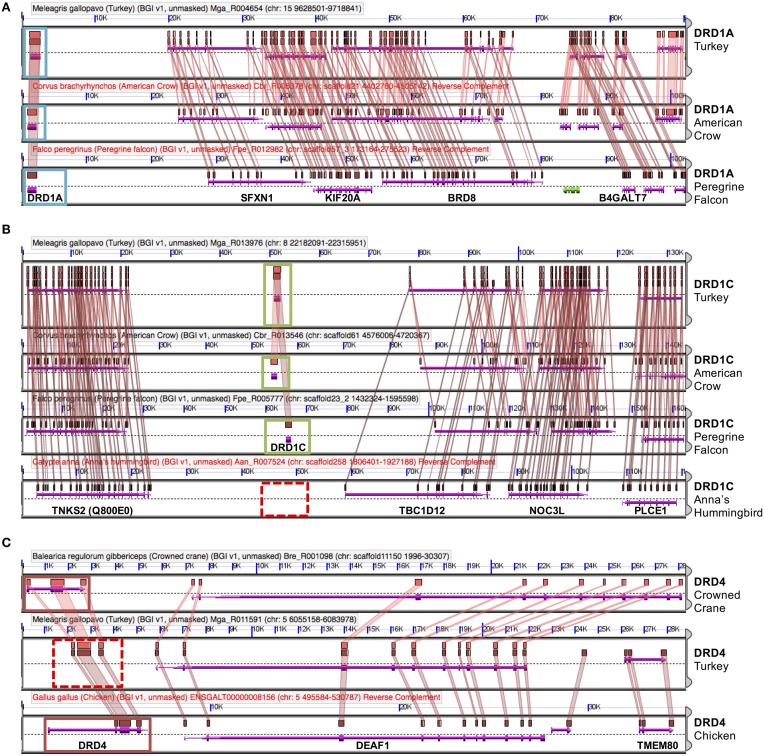
**Dopamine receptor identification by synteny**. Genes were confirmed via microsynteny analysis with known genes from Turkey or Chicken. Each comparison consists of genomic panels with the dashed line separating top and bottom strands of DNA. Gene models are represented by colored arrows above and below that dashed line, with gene symbols annotated on the bottom panel. Colored boxes drawn above gene models with lines connecting them between two genomic regions denote pair-wise regions of sequence similarity. Genes overlapped by regions of sequence similarity are colored purple; a colinear pattern of these genes is used to infer synteny. Analyses identified three situations: **(A)** Example of DRD1A confirmation, in American Crow and Peregrine Falcon. Confirmed genes are boxed in light blue. Note intact gene model (purple arrow), sequence similarity of genes, as well as collinear order of genes on surrounding genomic region. **(B)** Example of a missing gene (DRD1D), in Anna's Hummingbird. Syntenic genomic regions containing DRD1D are boxed in green and putatively absent DRD1D are indicated by a dashed red box. Note that there is no sequence similarity between syntenic regions where the gene should be present in hummingbird. **(C)** Example of a likely present but unannotated gene (DRD4) in Turkey. Solid red boxes indicate two present genes, and the dashed red box indicates the area with a missing gene model. Note the high level of sequence similarity between syntenic regions overlapping the missing gene (colored boxes with connecting colored wedges). Analyses can be regenerated at:**(A)** http://genomevolution.org/r/e7sj **(B**) http://genomevolution.org/r/e84r **(C**) http://genomevolution.org/r/e85v.

Not all receptors could be identified in the genome assemblies of all species of birds (Figure 2), suggesting losses could have occurred in some lineages. To test and confirm this possibility, we performed additional BLAST searches against the raw genomic sequencing reads for each missing receptor to ensure that receptor identification was not hampered by incomplete assembly of the genome in current builds (examples in Figure 3, Table 1, Supplementary File 1).

For the D1 DA receptors, DRD1A was successfully identified and classified in all investigated species (Figure 2, closed circles). DRD1B was identified in the genome builds of all species except White-Tailed Eagle. However, microsynteny alignments showed a scaffold (#35946) that contained a section of DRD1B coding sequence on its terminal end, indicative that the gene was not fully assembled following genome sequencing (Figure 2, open circle). Additionally, when DRD1B receptor coding sequences from Nightjar, Peregrine Falcon, Northern Fulmar, Medium Ground Finch, and Turkey were used as BLAST queries against the raw genomic sequencing reads from White-Tailed Eagle, matching BLAST hits covered almost the entire length of the query sequences (Table 1, Supplementary File 1). Thus, the White Tailed Eagle DRD1B is present, but lacking in annotation due to an incomplete genome assembly. DRD1C was not identified in the genome assembly of three distantly related species, golden collared manakin, great black cormorant, and Anna's hummingbird. Microsynteny analyses and BLAST searches of the raw sequencing reads suggested DRD1C was truly absent in these species (Figure 2, no circle; Table 1, Supplementary File 1). DRD1E was not found in the genome assembly builds of 7 of the 43 species, but only one (parakeet/budgerigar) appeared as a genuine loss (Figure 2, open circles; Table 1, Supplementary File 1). Overall, DRD1A and DRD1B were present in all species, while DRD1C and DRD1E were lost in some lineages independently. Notably, D1 receptor losses appear to correspond to the fastest diverging receptors, DRD1C and DRD1E (Figure 1B).

For D2 DA receptors, DRD2 and DRD3 were present in the genome builds of all avian species, but we were unable to find the DRD4 receptor in the genome builds over half of the species. However, microsynteny analysis and BLAST searches of the raw sequencing reads showed fragments of DRD4 were present in all the remaining half of the species, either in unannotated assembled regions or in unassembled regions (Figures 2, 3C, Supplementary File 1). These absences were not limited to low coverage genomes, and included high-coverage, well-assembled genomes with scaffold N50s > 1Mb [medium ground finch, Dalmatian pelican, hoatzin, Anna's hummingbird, Chimney swift, pigeon, duck, and ostrich (Zhang et al., 2014)]. However, coverage and genome N50 appears to contribute to these findings, as 17 of 21 (81%) of low coverage/N50 < 1 Mb genomes had unassembled DRD4 reads, whereas only 7 of 22 (32%) of high coverage/N50 > 1 Mb genomes had unassembled reads. Similar to DRD1C and DRD1E in the D1 receptors, DRD4 has the fastest diverging sequence of the D2 receptors, indicative of relaxed selective pressure on the gene. Combined, this evidence suggests the DRD4 receptor has a genomic sequence that is difficult to assemble and structurally annotate, and that it is likely that the gene is being psuedogenized and independently lost in many of the bird species.

### D1 and D2 vertebrate dopamine receptor families expanded through whole genome duplication

**Figure 4:**
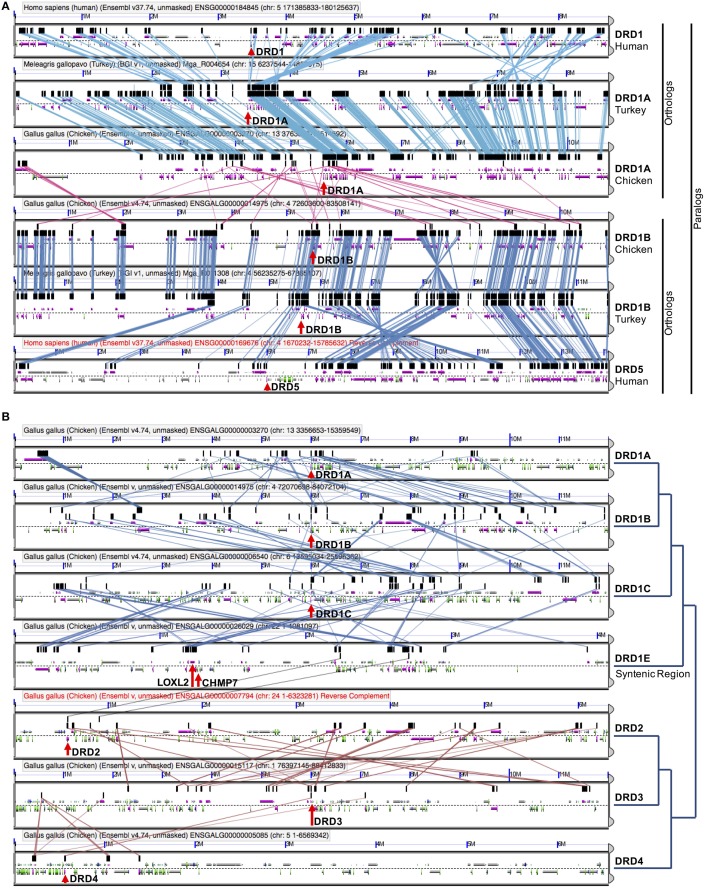
**DA receptor microsynteny analysis. (A)** Visualization of shared microsynteny between human, chicken, and turkey chromosome regions containing DRD1A (human DRD1) and DRD1B (human DRD5) receptors. Strong synteny can be seen between Turkey/Chicken DRD1A receptors and Turkey/Chicken DRD1B receptors. Human chromosome regions containing DRD1 and DRD5 receptors show clear synteny with respective orthologs containing chicken and turkey chromosome regions. Chicken DRD1A and DRD1B regions show synteny with one another, although the signal is weaker than between orthologous receptors in different species. **(B)** Microsynteny visualizations (left) and phylogenetic relationships (right) of all DA receptors in Chicken. Microsynteny can be observed between D1 receptors, with the strongest between DRD1A and DRD1B. No pattern of microsynteny is seen between the two classes of receptors, with weak synteny seen between DRD2 and DRD3. Details on how to read these microsynteny analyses are described in Figure 3. Results can be regenerated at **(A)** https://genomevolution.org/r/ho6d and **(B)** http://genomevolution.org/r/hjp0.

As expected, genomic regions containing each receptor showed strong synteny with regions from other species that contained the orthologous receptor (e.g., Figures 3A, 4A). Interestingly, the genomic regions containing the D1 receptors also showed weak but persistent synteny signals with the genomic regions that contained the other D1 receptors (strongest between DRD1A/DRD1B), both within and across species (Figure 4, Table 2). A similar pattern of intragenomic synteny evidence was observed between chromosome regions containing DRD2 and DRD3 (Figure 4B, Table 2). This synteny signal indicated that these genomic regions, and thus these receptor sets, might have originated from a common ancestor by duplication of large chromosomal regions (e.g., polyploidy). This pattern was not observed between any Class 1 and Class 2 receptors, or between the other D2 receptor pairs (Figure 4B).

**Table 2:** **Duplication evidence summary**.

	**Microsynteny**		**SynFind**		**SynMap**	**Human Paralogy**
	**2 MB**	**12 MB**	**Colinear**	**Density**		
**DRD1A/DRD1B**	Y	Y	Y	Y	Y	Y
**DRD1A/DRD1C**	Y	Y	Y	Y	Y	N
**DRD1A/DRD1E**	Y	Y	Y	Y	N	N
**DRD1B/DRD1C**	Y	Y	N	Y	Y	N
**DRD1B/DRD1E**	Y	Y	N	Y	N	N
**DRD1C/DRD1E**	Y	Y	N	N	N	N
**DRD2/DRD3**	N	Y	N	Y	N	Y
**DRD2/DRD4**	N	N	N	N	N	N
**DRD3/DRD4**	N	N	N	N	N	N

*Evidence supporting a WGD origin of each DA receptor pair. For the D1 receptors, evidence follows a pattern consistent with fractionation loss across all D1 receptors, but is only conclusive for DRD1A/DRD1B. For D2 receptors, evidence suggests DRD2/DRD3 arose from a WGD, but the evidence is weaker than for DRD1A/DRD1B. In no cases was evidence for duplication between classes D1 and D2 receptors found (not shown). Microsynteny analyses were performed with GEvo for two genomic windows: 2 MB and 12 MB. SynFind analyses were performed using both collinear and density based scoring algorithms, with a gene window size of 40 and minimum number of four syntenic genes. SynMap analyses aimed to identify whether gene pairs were located in chromosome regions with evidence of a WGD. GEvo, SynFind, and SynMap are all CoGe tools, are available online at: http://www.genomevolution.org (Lyons and Freeling, 2008). Human paralogy analyses (e.g., **Figure 7**) are based on McLysaght et al. (2002) and aimed to identify whether the human orthologs of gene pairs fell in regions shown as statistically likely as having duplicated from a WGD event. In all analyses, Y indicates evidence was found, and N indicates a lack of evidence*.

**Table 3:** **Reference receptor accessions**.

**Species**	**Gene name**	**Accession ID**
Chicken	DRD1A	ENSEMBL: ENSGALG00000003270
Chicken	DRD1B	ENSEMBL: ENSGALG00000014975
Chicken	DRD2	ENSEMBL: ENSGALG00000007794
Chicken	DRD3	ENSEMBL: ENSGALG00000015117
Chicken	DRD4	ENSEMBL: ENSGALG00000005085
Turkey	DRD1A	ENSEMBL: ENSMGAG00000015442
Turkey	DRD1B	ENSEMBL: ENSMGAG00000012700
Turkey	DRD2	ENSEMBL: ENSMGAG00000004223
Turkey	DRD3	ENSEMBL: ENSMGAG00000014321
Turkey	DRD4	ENSEMBL: ENSMGAG00000003742
Zebra Finch	DRD1A	ENSEMBL: ENSTGUG00000000340
Zebra Finch	DRD1B	ENSEMBL: ENSTGUG00000009908
Zebra Finch	DRD2	ENSEMBL: ENSTGUG00000000255
Zebra Finch	DRD3	ENSEMBL: ENSTGUG00000013405
Zebra Finch	DRD4	ENSEMBL: ENSTGUG00000007173
Human	DRD1	ENSEMBL: ENSG00000184845
Human	DRD2	ENSEMBL: ENSG00000149295
Human	DRD3	ENSEMBL: ENSG00000151577
Human	DRD4	ENSEMBL: ENSG00000069696
Human	DRD5	ENSEMBL: ENSG00000169676
Mouse	DRD1A	ENSEMBL: ENSMUSG00000021478
Mouse	DRD1B	ENSEMBL: ENSMUSG00000039358
Mouse	DRD2	ENSEMBL: ENSMUSG00000032259
Mouse	DRD3	ENSEMBL: ENSMUSG00000022705
Mouse	DRD4	ENSEMBL: ENSMUSG00000025496
Fugu	DRD2	ENSEMBL: ENSTRUG00000014690
Fugu	DRD3	ENSEMBL: ENSTRUG00000000584
Fugu	DRD4	ENSEMBL: ENSTRUG00000012466
Lamprey	DRD1A/B	ENSEMBL: ENSPMAG00000010421
Ciona	D1-Like	GenScript: XM_004226475
Drosophila	Dop1 (D1-like)	Flybase: FBgn0011582

*Accession numbers for receptors sequences obtained from external resources. Coding sequence for each receptor was obtained from the listed databases*.

**Figure 5:**
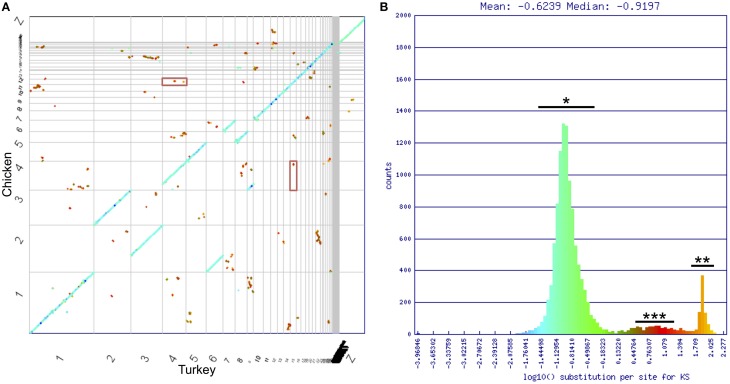
**Chicken/turkey synonymous dot plot. (A)** Syntenic dot plot of Chicken (Y-axis) and Turkey (X-axis) genomes. Horizontal or vertical gray lines delineate chromosomes. Each dot represents an orthologous gene pair. Dots are colored based on synonymous mutation rate (Ks) as calculated between each orthologous gene pair. **(B)** Histogram of log_10_ transformed Ks values. Three major groupings of Ks values can be identified. Blue/Green is typical of orthologous genes derived from the divergence of lineages (^*^). Orange is algorithmic noise (^**^; *Ks* values >80 substitutions per synonymous site). Small red group is indicative of an ancient whole genome duplication event (^***^). DRD1A/DRD1B genes fall within this latter cluster, and are shown in the red boxes in **(A)**. Analysis can be regenerated at https://genomevolution.org/r/drgh.

To uncover more information on the nature of these possible duplications, we used SynMap (Lyons and Freeling, 2008) to generate a syntenic dot plot of the Chicken and Turkey genomes, with syntenic regions identified where 4 colinear genes fell within a 40 gene window. Synonymous mutation values (Ks) were calculated for each identified syntenic gene pair, and a color scheme was used to illustrate the values on the plot (Figure 5A). Ks values are frequently used as a relative molecular clock, since higher values are indicative of more neutral mutations having occurred and hence more time since divergence from a common ancestor (Kreitman and Akashi, 1995). The majority of syntenic regions occurred within three distinct clusters of synonymous mutation values (Figure 5B). The largest cluster (^*^) contained the orthologous syntenic gene pairs that derived from the divergence of two species. These genes share an average log_10_ Ks value of ~–0.8 (~0.16 synonymous mutations per synonymous site). The smallest cluster (^***^) appeared to have also originated contemporaneously. The high substitution values (~10 synonymous mutations/synonymous site) of this second cluster, along with the genome-wide scattering of the orthologous regions (Figure 5A) follow a pattern consistent with an ancient whole genome duplication (WGD) event (Blanc et al., 2003; Blanc and Wolfe, 2004). The remaining cluster (^**^) had a log_10_ Ks value = ~1.9 (~80 synonymous mutations/synonymous site), which is consistent with algorithmic noise (Tang and Lyons, 2012).

Of the D1 receptors, gene pairs DRD1A/DRD1B were located in regions identified by SynMap with evidence of originating through a WGD, DRD1A/DRD1C, and DRD1B/DRD1C were located near, but not within such regions, and WGD evidence was not present in regions containing DRD1E (Figure 5, Table 2). This pattern of conservation is consistent with the fractionation of homeologous (partially homologous) gene content that follows polyploidy, as has been shown in plant systems (Thomas et al., 2006; Schnable et al., 2011; Wang et al., 2011; Cheng et al., 2012; Sankoff and Zheng, 2012). For D2 receptors, no gene pairs were located in regions identified by SynMap with evidence to support a WGD. However, when a “density” based algorithm (4 syntenic genes within a 40 gene window, order disregarded) was used instead of the colinear algorithm to determine syntenic gene regions, genomic regions containing DRD2 and DRD3 showed a signal of synteny (Table 2). This suggest that while the colinear arrangement of syntenic genes was lost around DRD2 and DRD3, the overall regions are indeed syntenic (Wolfe, 2001). The DRD4 containing region did not show evidence of synteny with the other D2 receptor regions and no pair of D1/D2 genes showed synteny evidence, under either colinear or density based algorithms.

**Figure 6:**
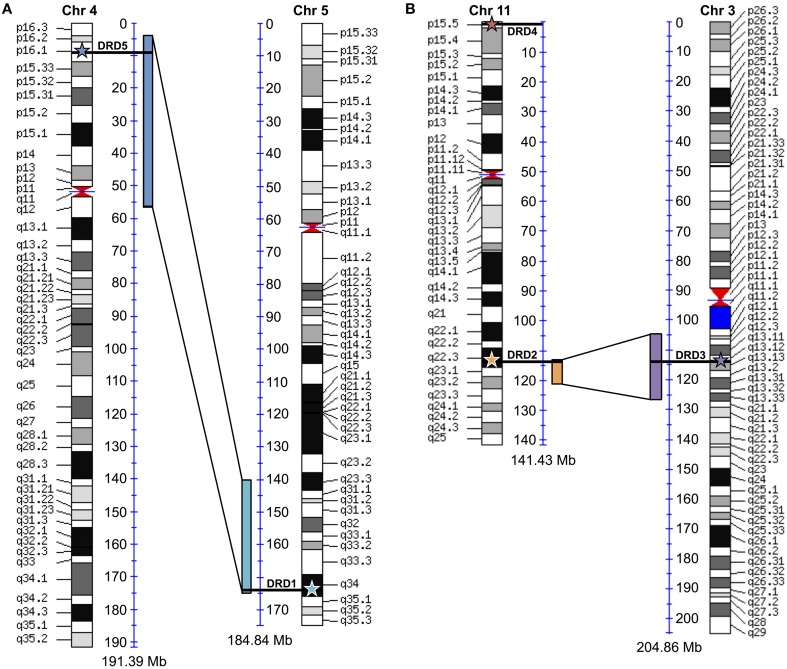
**Human paralagon maps**. Human chromosome maps showing regions previously classified as likely having originated from a whole genome duplication event in vertebrate history. **(A)** Human DRD1 (light blue star) and DRD5 (dark blue star) are marked on their respective chromosomes, and can be seen to fall within these regions. **(B)** Human DRD2 (orange star), DRD3 (purple star), and DRD4 (red star) mapped on their respective chromosomes. DRD2/DRD3 fall within these regions. Maps were generated using “Paralogons in the Human Genome” tool from the Wolfe lab, and are based on a minimum threshold for paired proteins per block greater than 12 **(A)** or 7 **(B)**. Paired proteins per block greater than 6 are statistically probable as having occurred from a WGD event rather than by chance (McLysaght et al., 2002). Chromosome maps can be regenerated and viewed at http://wolfe.ucd.ie/cgi-bin/dup_528/chrom_plot.

Additional evidence for the WGD origin of the DRD1A/DRD1B and DRD2/DRD3 receptors was sought through comparative analysis with the human genome, where we investigated whether the human orthologs of the avian genes were located in chromosome regions previously identified as likely derived from an ancient vertebrate WGD (McLysaght et al., 2002). To confirm previous assertions that human DRD1 (hDRD1) and hDRD5 are direct orthologs to the avian DRD1A and DRD1B receptors, respectively, a microsynteny analysis of genomic regions containing these genes between Human, Turkey, and Chicken was performed (Figure 4A). In this analysis, it was clear that hDRD1 and avian DRD1A are orthologous, and hDRD5 and avian DRD1B are also orthologous. Similar analyses revealed that the three D2 receptors in Chicken and Human were all orthologous between species (not shown). hDRD1, hDRD5, hDRD2, hDRD3, and hDRD4 were all mapped onto human chromosome plots marked with regions demonstrated by McLysaght et al. (2002) as statistically likely to have originated from an early vertebrate WGD (Figure 6). hDRD1/hDRD5, as well as hDRD2/hDRD3, fell within these regions. Since the human orthologs of avian DRD1C and DRD1E have been lost in the mammalian lineage and could not be directly compared, we aligned human chromosome regions syntenic to the avian regions containing DRD1C and DRD1E, identified where the genes were most likely located prior to loss, and plotted these locations onto human chromosome maps. Gene regions containing DRD1, DRD5, and those syntenic to DRD1C and DRD1E fell in four regions believed to have been a linkage group quadruplicated in stem chordates, (Pébusque et al., 1998; Putnam et al., 2008; Yamamoto et al., 2013), but did not fall directly within regions shown by McLysaght et al. (2002) to be statistically likely as having originated from a WGD (Table 2). No pair of human D1/D2 receptors fell within these regions, and hDRD4 did not fall in a region with the other human D2 receptors.

### Evolutionary analysis of DRD1A/DRD1B duplication in the vertebrate linage

**Figure 7:**
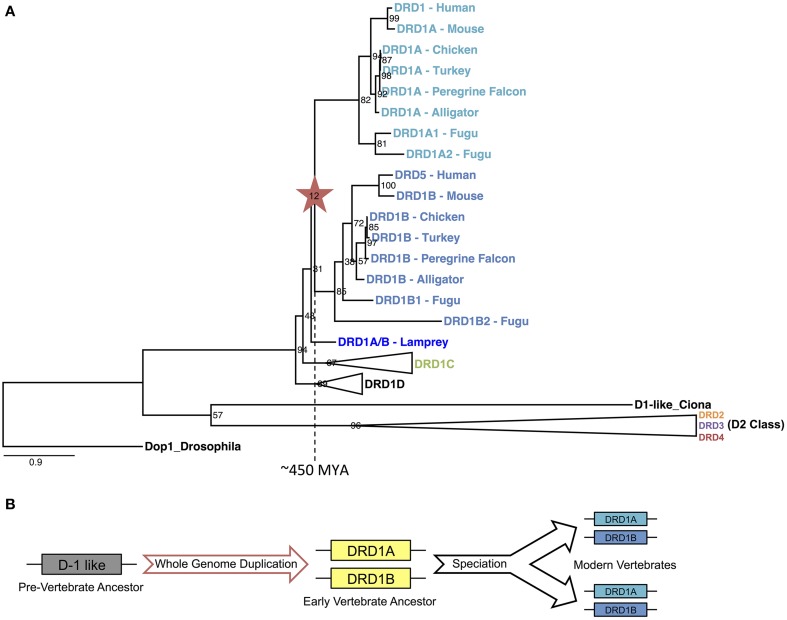
**DRD1A/DRD1B evolutionary phylogeny and proposed model. (A)** Phylogenetic relationship of D1 and D2 dopamine receptors for two invertebrate (Drosophila and Ciona D1-like) and seven vertebrates (Lamprey, Fugu, Alligator, Turkey, Chicken, Mouse, and Human) species based on protein coding sequences. Bootstrap support values are shown at nodes; branch lengths (substitutions/changes per site) are shown proportionally. DRD1C, DRD1D, and all D2 clades are collapsed for clarity, but expanded clades may be found in Supplemental File 3: Figure S5. Note that fugu has an independent whole genome duplication. **(B)** Illustrated model of DRD1A/DRD1B evolution, based on events inferred from the evolutionary phylogeny.

To determine if the WGD that gave rise to the DA receptor expansion was specific to the vertebrate lineage, we constructed a phylogeny of DA receptors across a range of vertebrate and invertebrate species rooted on an arthropod, drosophila (Figure 7A). As expected, each family of receptors formed distinct clades, each monophyletic for that family. Using our methods for gene identification, duplicates of each D1 receptors DRD1A, DRD1B, and DRD1C were identified in fugu (Figure 7A, Supplemental File 3). These duplications are likely explained by an additional WGD that occurred in the fugu lineage (Panopoulou and Poustka, 2005). With regards to DRD1A/DRD1B duplication, the phylogeny showed a clear transition in the vertebrate lineage, with a WGD event near the origin of vertebrates approximately 450 MYA (Figure 7A) (Dehal and Boore, 2005).

## Discussion

Understanding DA and its associated receptors is critical for both health and agricultural sciences due to the significant roles they play in in behavior, learning, and general neuromodulation. In this study we aimed to expand our understanding of both the recent evolution of DA receptors across the avian lineage as well as the ancient evolution of DA receptors throughout the larger vertebrate lineage. Using a combination of phylogenetic and sequence analyses, we identified and classified the DA receptors in 43 recently sequenced bird species spanning the diversity of extant avian species. Our analyses show that the ancestral bird genome contained at least 7 DA receptors: four D1 receptors (DRD1A, DRD1B, DRD1C, and DRD1E) and three D2 receptors (DRD2, DRD3, and DRD4). Two of the D1 receptors (DRD1A, DRD1B) and two of the D2 receptors (DRD2 and DRD3) were maintained across all extant species. The other two D1 receptors, DRD1C and DRD1E, have been lost in some lineages independently, although they are still widely conserved across the lineage. The remaining D2 receptor DRD4 has been either pseudogenized and thus functionally lost in many species, or has increased sequence complexity making it difficult to computationally identify, assemble, and annotate.

When receptor losses are compared to the species phylogeny, no apparent species-level pattern of gene loss can be identified. With regards to the Class 1 receptors, these findings strongly support earlier claims that DRD1C and DRD1E receptors can be more easily lost in multiple vertebrate lineages independently (Yamamoto et al., 2013). One interesting observation is that these two receptors that have undergone loss are diverging 2–4 times faster than the retained receptors DRD1A and DRD1B. Similarly, the fastest diverging D2 receptor, DRD4, appears to have been lost independently from species spanning the avian species tree. This pattern of loss of the more-rapidly diverging receptors may suggest relaxed functional constraints due to overlapping function, allowing some receptors to undergo modification (e.g., neofunctionalization) and/or loss without negative impacts in those species. The possible pseudogenization of DRD4 in many bird species is intriguing, considering that variations in the coding sequence of the gene are correlated with creativity and other personality differences within a species, including in birds and humans (Fidler et al., 2007; Ben-Israel et al., 2015). If future studies confirm that DRD4 is indeed being pseudogenized and lost in many lineages, it would be interesting to perform additional experiments in species that do not have a DRD4 receptor to determine possible effects on personality traits, and whether other dopamine receptors functionally replace it. Detailed expression studies of the location and timing of these genes should shed further light into this possibility.

In terms of evolutionary history of DA receptors, while there has been long-standing speculation that D1 receptors DRD1A and DRD1B could have originated from a WGD event, conclusive evidence has not been provided (Holland, 1999; Le Crom et al., 2003). Through microsynteny, genome-wide synteny, synonymous substitution metrics, phylogenetic, and comparative genomic analyses of many genomes, we provide structural and temporal evidence that DRD1A and DRD1B, as well as DRD2 and DRD3 receptors originated through WGD.

We hypothesize that the weaker colinear signal of homologous genes from the genomic regions containing DRD2 and DRD3 could be due to the diploidization process following polyploidy, where many duplicated homeologous genes are lost through a process known as fractionation, relatively quickly reducing the gene content of a genome to a state more similar to the pre-duplicated ancestral genome (Woodhouse et al., 2010). In addition to fractionation, larger structural rearrangements, including chromosome fissions, fusions, translocations, and local inversions may also occur causing further obfuscation of the colinear signal of retained duplicated genes, which has been seen in vertebrate lineages (Smith et al., 2002). This difference in synteny signal strength is interestingly correlated with a single exon D1 receptor (i.e., low recombination) and multi-exon D2 receptors. However, despite the surrounding gene losses and local rearrangements in D2 receptor chromosome regions, we find that many duplicated genes around the receptors are retained, and a signal of synteny can still be identified.

Yamamoto et al. (2013) proposed an evolutionary model for the origin of the DRD1 receptors where an ancient duplication gave rise to the ancestor of DRD1A/B and DRD1C/E. While our data clarifies the mechanism and origin of DRD1A and DRD1B, it is not conclusive on the origin of DRD1C and DRD1E sharing an ancestor to the exclusion of DRD1A/B. Similar to the results they present, our phylogeny of D1 receptors (Figure 1) shows a polytomy for DRD1E even though there is syntenic evidence for their orthologous relationship across the sampled avian taxa, which is likely a result of this family's rapid divergence rate. In addition, syntenic analysis of DRD1C and DRD1E shows weak support for being derived from WGD, but not to a great extent when compared pairwise with DRD1A and DRD1B (Table 2). Nevertheless, we believe the data and methods we present, when combined with additional data and comparative analyses, will aid future studies to resolve the exact relationships and origins of DRD1C and DRD1E receptors.

In summary, our evidence suggests a large scale, retained expansion of the DA receptor family through an early vertebrate WGD event, followed by subsequent losses of the more rapidly diverging receptors in some species lineages independently, suggesting relaxed functional constraints on some receptors in the DA receptor superfamily. We propose that the early vertebrate ancestor possessed only one D1 receptor. An ancient WGD, likely one of the vertebrate-lineage-specific 1R or 2R events (Dehal and Boore, 2005; Panopoulou and Poustka, 2005), gave rise to the DRD1A and DRD1B receptors approximately 450 MYA (Figure 7B). An additional round of WGD or translocation duplication events may have given rise to the additional D1 receptors DRD1C/D and DRD1E, but fractionation complexities of layered polyploidy events mask conclusive evidence (Le Crom et al., 2003; Thomas et al., 2006; Sankoff and Zheng, 2012). In regards to the D2 receptors, our evidence suggests that DRD2 and DRD3 also originated from an early WGD, but DRD4 originated from a smaller scale duplication event. Our evidence provides the first conclusive support for a long-standing hypothesis of dopamine receptor evolution, and also suggests that the expansion of dopamine receptors through WGD was greater than previously expected. These findings shed light into the evolutionary relationships of a gene family important in brain function and will hopefully help unravel complex functional relationships between similar but distinct neuroregulatory receptors.

### Conflict of interest statement

The authors declare that the research was conducted in the absence of any commercial or financial relationships that could be construed as a potential conflict of interest.

## Footnotes

^1^GenScript - Make Research Easy - The leader in molecular cloning and gene synthesis, peptide synthesis, protein and antibody engineering. Available online at: http://www.genscript.com/ (Accessed December 19, 2014).
