# Supplementary material for: Identification of dopamine receptors across the extant avian family tree and analysis with other clades uncovers a polyploid expansion among vertebrates
Source: Front Neurosci. 2015 Oct 7;9:361. doi: 10.3389/fnins.2015.00361 (PMC4595791; doi:10.3389/fnins.2015.00361)

Supplementary File 1

Missing Receptor Analysis

Detailed microsynteny analyses and BLAST searches against raw sequencing reads for unidentified receptors. Organized by species. All raw reads can be accessed through the NCBI BioProject for each species (See Table 1)

Reading Guide

All missing receptors were analyzed with microsynteny analyses and BLAST searches against the raw reads to determine if genes were truly absent or if they were just unidentifiable with our methods.

1. Microsynteny Analyses

    Microsynteny analyses of the region that should contain the missing genes are compared with a known species (turkey or chicken). Each panel represents a genomic region, with a dashed line separating the top and bottom strands of the chromosome. Gene models are drawn as colored arrows. Regions of sequence similarity are represented by colored boxes, which are connected between panels to show patterns of similarity and synteny. Orange shading indicates gaps in sequences. The known receptor is indicated by a blue box in the top panels. For more information on reading GEvo panels, see *https://www.genomevolution.org/wiki/index.php/GEvo*.

    Receptors are classified as "unidentified" if sequencing gaps appear in region where gene should be found (example: Zebra Finch DRD1E), if scaffold ends would split the gene model (example: Rifleman DRD4), or if GenomeThreader predicts a gene model, but no annotation is present in the assembly (example: Medium Ground Finch DRD4).

2. Raw Read BLAST Searches

    BLAST searches were performed on the raw sequencing reads from each species where receptors were unidentified. For unidentified receptors, a multi-FASTA consisting of five known gene model sequences was used as the query. Read coverage is shown for each missing receptor. To serve as a control, the endogenous DRD1A (present in all species) sequence was used as a query. Where DRD1A did not return significant coverage, raw reads are not appropriate for missing receptor analysis (example: Zebra Finch DRD1E).

    Receptors were classified as "absent" if microsynteny analyses did not indicate any problems with assembly, and BLAST searches of the raw reads returned low coverage of the query sequence.

1. Zebra Finch

Present: DRD1A, DRD1B, DRD1C, DRD2, DRD3, DRD4
Unidentified: DRD1E
Absent: N/A

1.1 Microsynteny Analysis

DRD1E

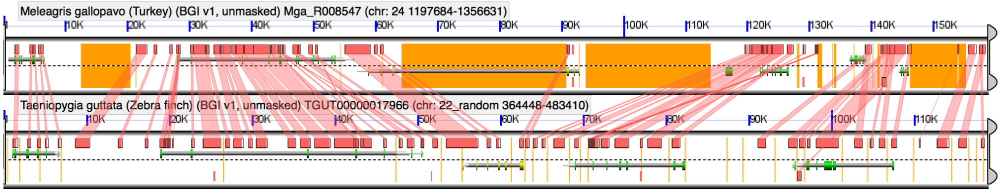

Regenerate Analysis: https://genomevolution.org/r/fiwq

1.2 Raw Reads Blast Results

DRD1A

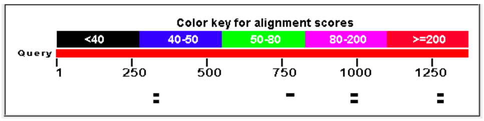

DRD1E

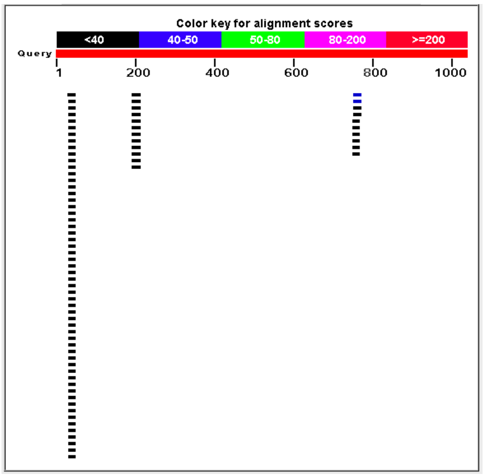

2. Medium Ground Finch

Present: DRD1A, DRD1B, DRD1C, DRD1E, DRD2, DRD3
Unidentified: DRD4
Absent: N/A

2.1 Microsynteny Analysis

DRD4

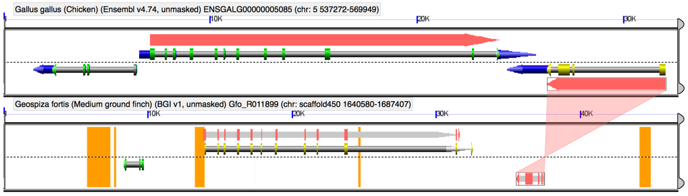

Regenerate Analysis: https://genomevolution.org/r/ffof

2.2 Raw Read BLAST Results

DRD1A

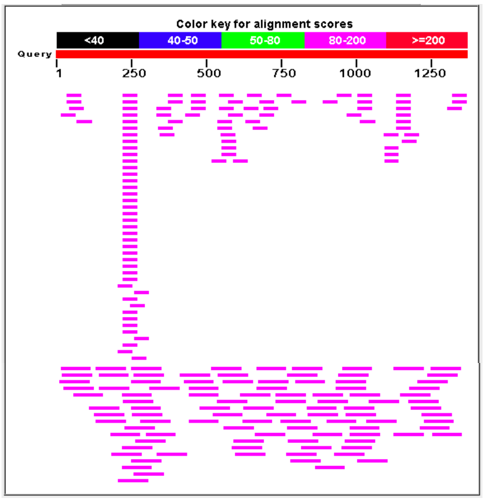

DRD4

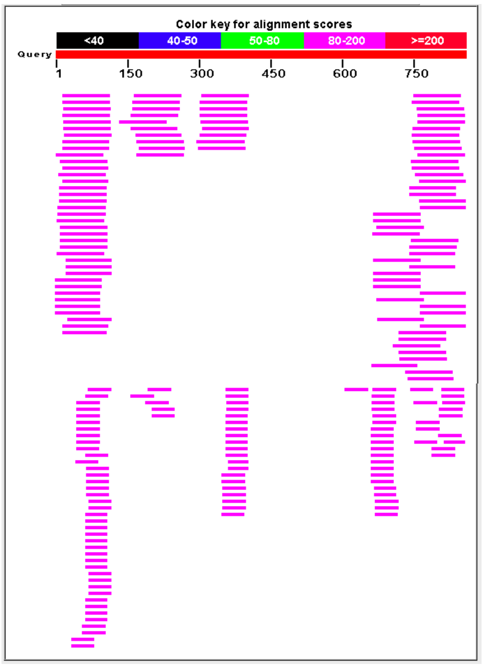

3. Golden Collared Manakin

Present: DRD1A, DRD1B, DRD2, DRD3, DRD4
Unidentified: DRD1E
Absent: DRD1C

3.1 Microsynteny Analysis

DRD1C

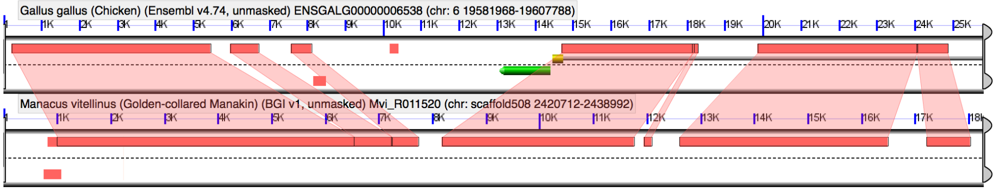

Regenerate Analysis: https://genomevolution.org/r/fms4

DRD1E

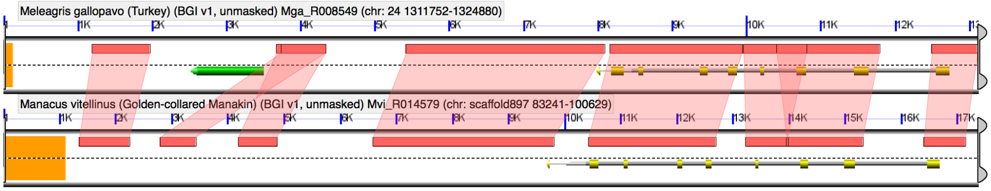

Regenerate Analysis: https://genomevolution.org/r/fms5

3.2 Raw Reads BLAST Results

DRD1A

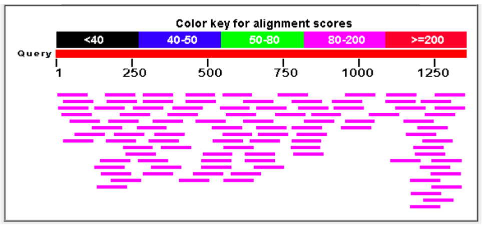

DRD1C

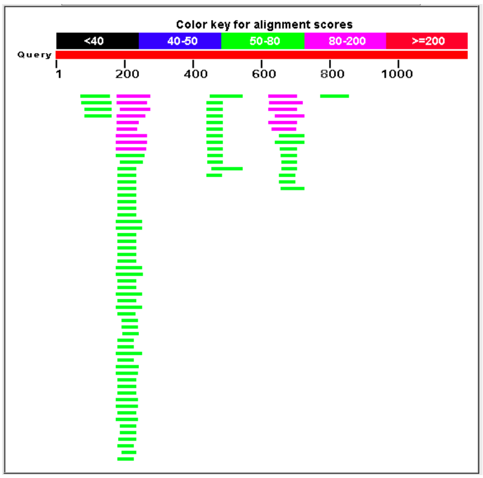

DRD1E

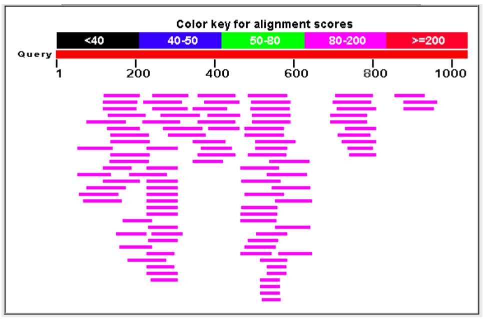
Color key for alignment scores
<40
40-50
50-80
80-200
>=200
Query
1
200
400
600
800
1000

4 Rifleman

Present: DRD1A, DRD1B, DRD1C, DRD1E, DRD2, DRD3
Unidentified: DRD4
Absent: N/A

4.1 Microsynteny Analysis

DRD4

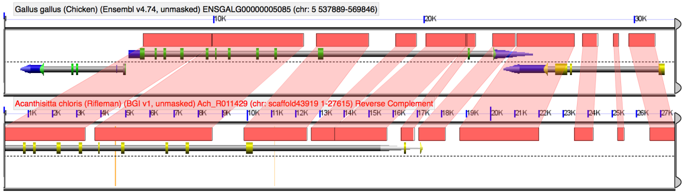

Regenerate Analysis: https://genomevolution.org/r/ffti

4.2 Raw Reads BLAST Results

DRD1A

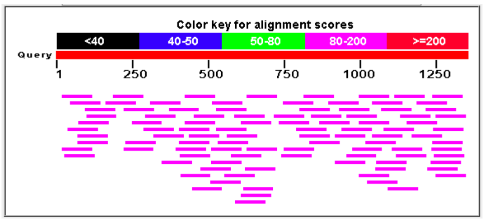

DRD4

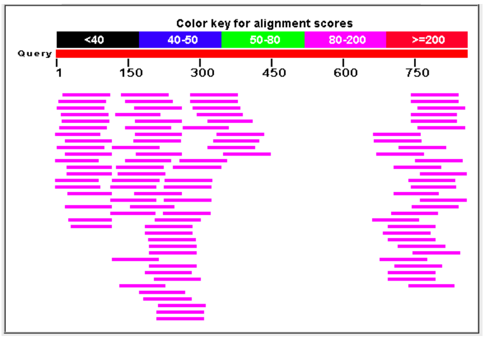

5. Budgerigar

Present: DRD1A, DRD1B, DRD1C, DRD2, DRD3, DRD4
Unidentified: N/A
Absent: DRD1E

5.1 Microsynteny Analysis

DRD1E

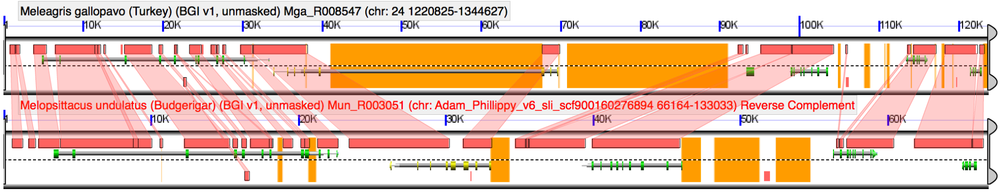

Regenerate Analysis: https://genomevolution.org/r/fiyl

5.2 Raw Reads BLAST Results

DRD1A

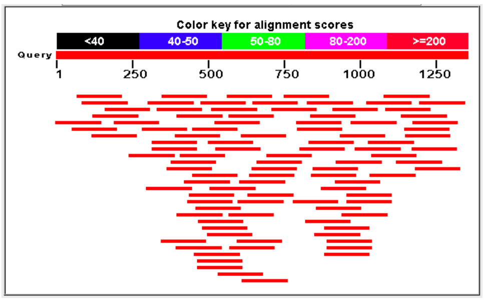

DRD1E

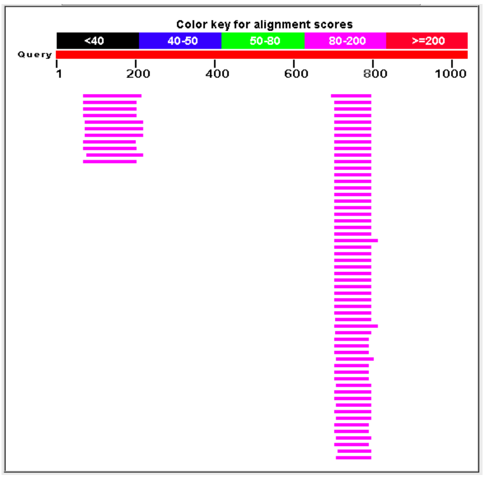

6. Red Legged Seriema

Present: DRD1A, DRD1B, DRD1C, DRD1E, DRD2, DRD3
Unidentified: DRD4
Absent: N/A

6.1 Microsynteny Analysis

DRD4

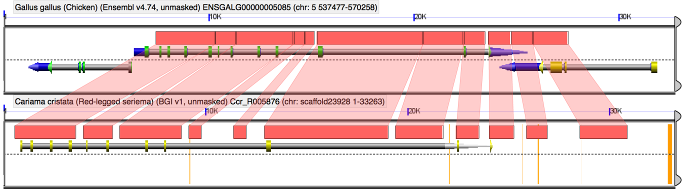

Regenerate Analysis: https://genomevolution.org/r/fftf

6.2 Raw Reads Blast Results

DRD1A

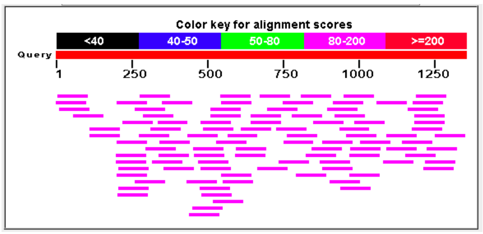

DRD4

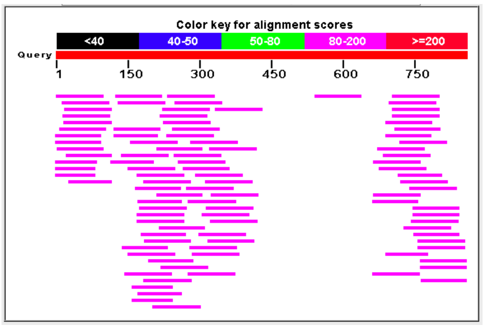

7. Northern Carmine Bee Eater

Present: DRD1A, DRD1B, DRD1C, DRD1E, DRD2, DRD3
Unidentified: DRD4
Absent: N/A

7.1 Microsynteny Analysis

DRD4

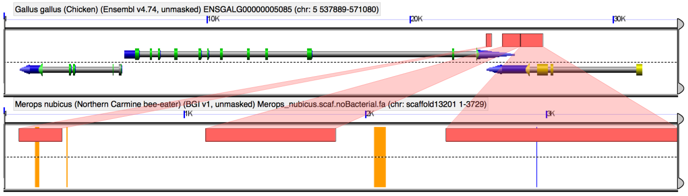

Regenerate Analysis: https://genomevolution.org/r/fftl

7.2 Raw Reads BLAST Results

DRD1A

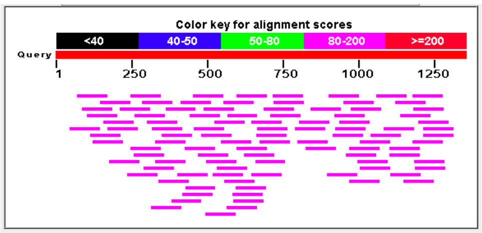

DRD4

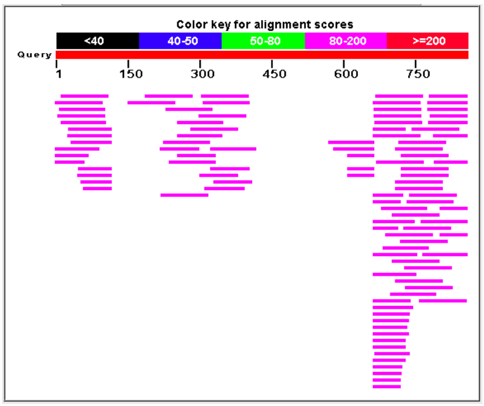

8. Javan Rhinoceros Hornbill

Present: DRD1A, DRD1B, DRD1C, DRD1E, DRD2, DRD3
Unidentified: DRD4
Absent: N/A

8.1 Microsynteny Analysis

DRD4

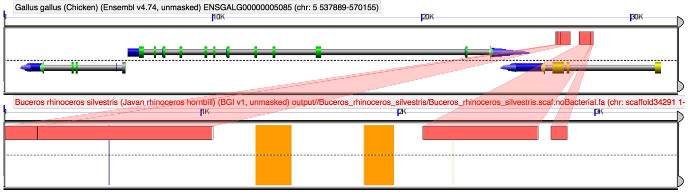

Regenerate Analysis: https://genomevolution.org/r/ffts

8.2 Raw Reads BLAST Results

DRD1A

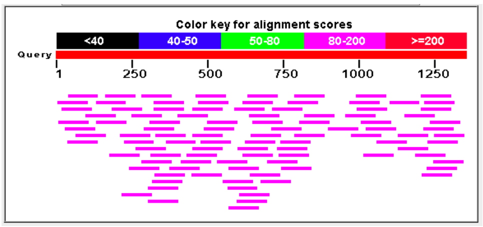

DRD4

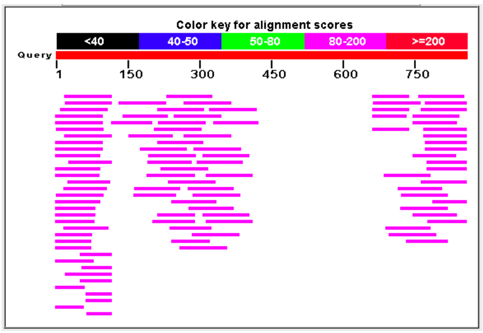

9. Bar Tailed Trogon

Present: DRD1A, DRD1B, DRD1C, DRD1E, DRD2, DRD3
Unidentified: DRD4
Absent: N/A

9.1 Microsynteny Analysis

DRD4

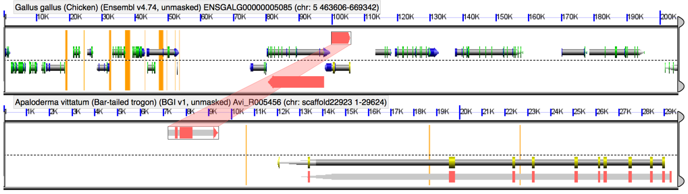

Regenerate Analysis: https://genomevolution.org/r/ffu2

9.2 Raw Reads BLAST Results

DRD1A

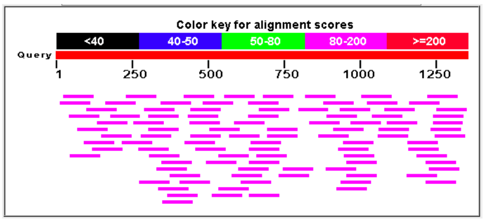

DRD4

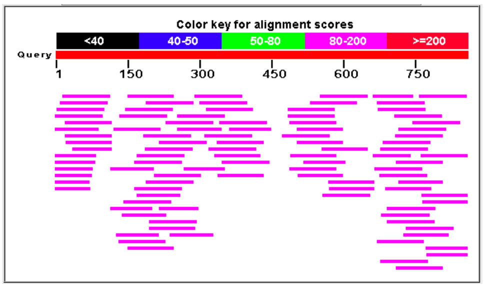

10. Speckled Mousebird

Present: DRD1A, DRD1B, DRD1C, DRD2, DRD3
Unidentified: DRD1E, DRD4
Absent: N/A

10.1 Microsynteny Analysis

DRD1E

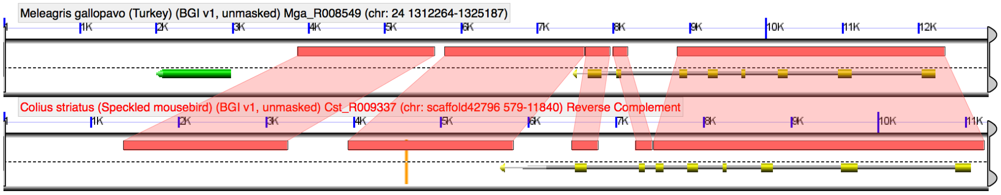

Regenerate Analysis: https://genomevolution.org/r/fj2m

DRD4

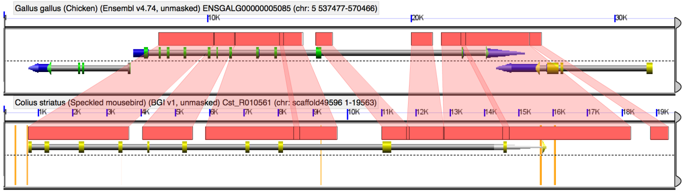

Regenerate Analysis: https://genomevolution.org/r/ffuf

10.2 Raw Reads BLAST Results

DRD1A

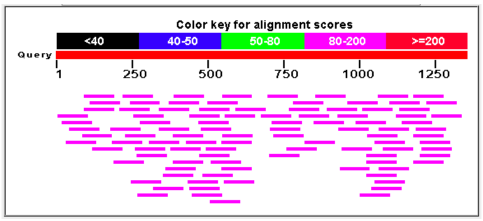

DRD1E

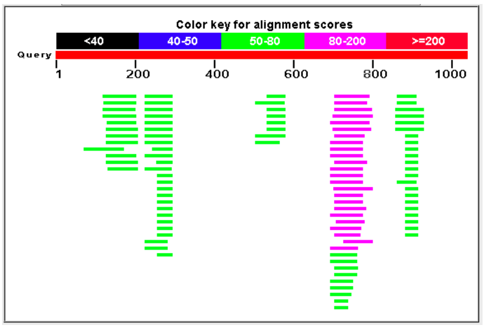

DRD4

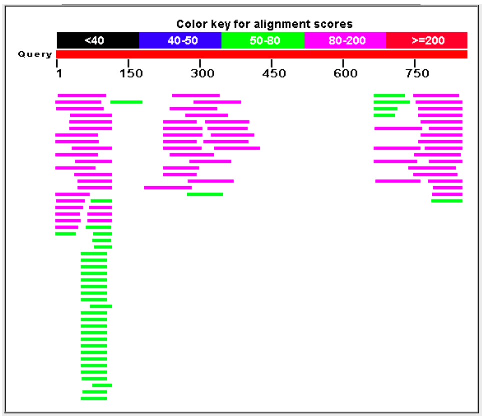
Color key for alignment scores
<40
40-50
50-80
80-200
>=200
Query
1
150
300
450
600
750

11. Barn Owl

Present: DRD1A, DRD1B, DRD1C, DRD1E, DRD2, DRD3
Unidentified: DRD4
Absent: N/A

11.1 Microsynteny Analysis

DRD4

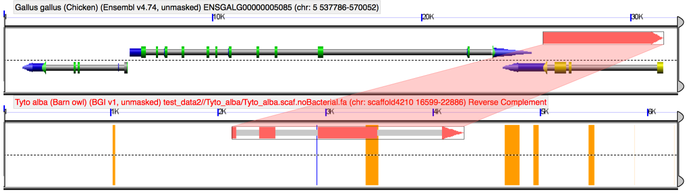

Regenerate Analysis: https://genomevolution.org/r/ffun

11.2 Raw Reads BLAST Results

DRD1A

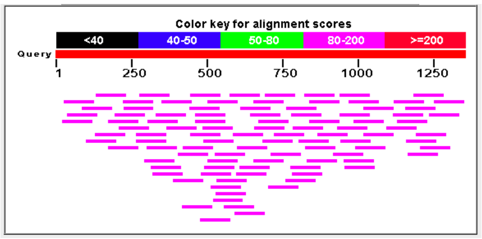

DRD4

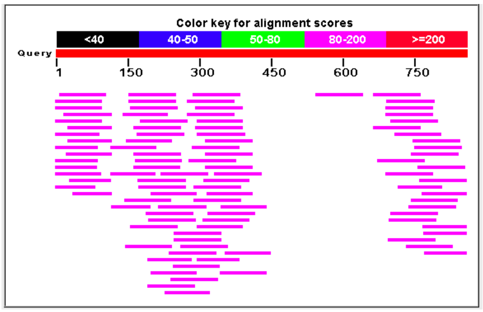

12. White Tailed Eagle

Present: DRD1A, DRD1C, DRD1E, DRD2, DRD3, DRD4
Unidentified: DRD1B
Absent: N/A

12.1 Microsynteny Analysis

DRD1B

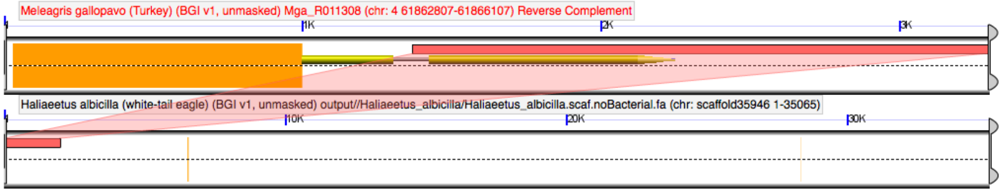

Regenerate Analysis: https://genomevolution.org/r/fncd

12.2 Raw Reads BLAST Results

DRD1A

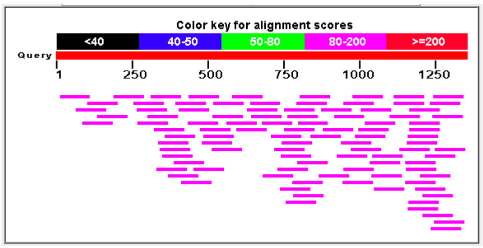

DRD1B

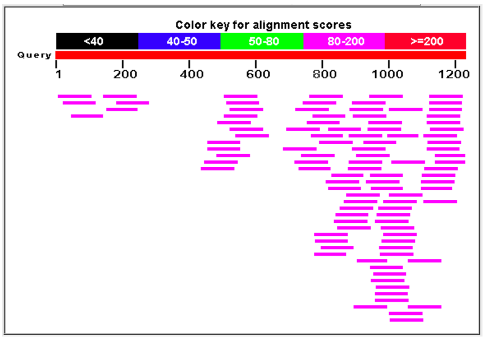

13. Dalmatian Pelican

Present: DRD1A, DRD1B, DRD1C, DRD1E, DRD2, DRD3
Unidentified: DRD4
Absent: N/A

13.1 Microsynteny Analysis

DRD4

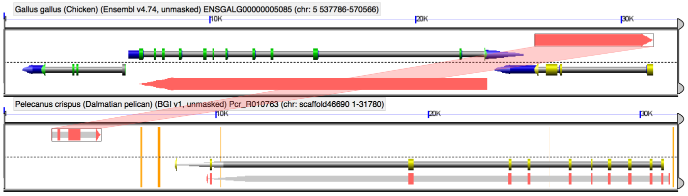

Regenerate Analysis: https://genomevolution.org/r/ffuu

13.2 Raw Reads BLAST Results

DRD1A

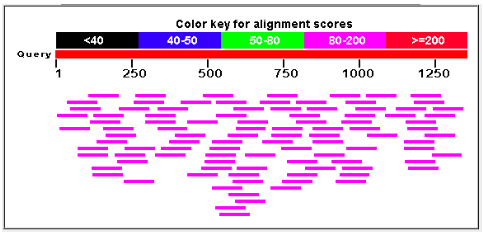

DRD4

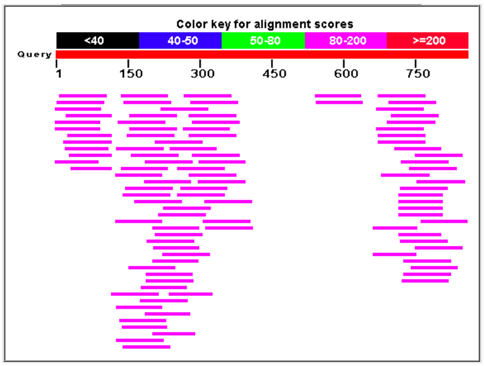

14. Great Black Cormorant

Present: DRD1A, DRD1B, DRD1E, DRD2, DRD3
Unidentified: DRD4
Absent: DRD1C

14.1 Microsynteny Analysis

DRD1C

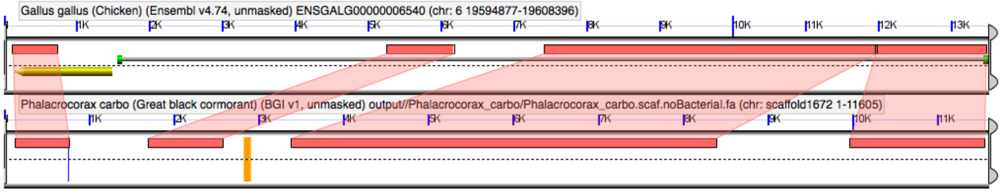

Regenerate Analysis: https://genomevolution.org/r/fgnm

DRD4

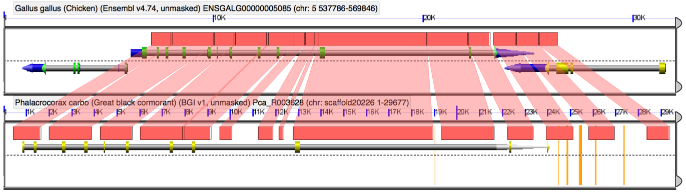

Regenerate Analysis: https://genomevolution.org/r/ffv2

14.2 Raw Reads BLAST Results

DRD1A

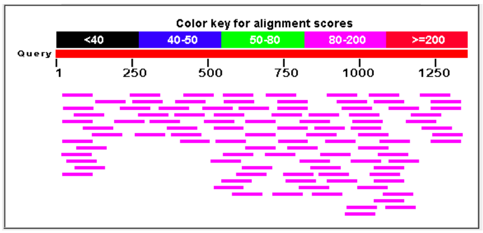

DRD1C

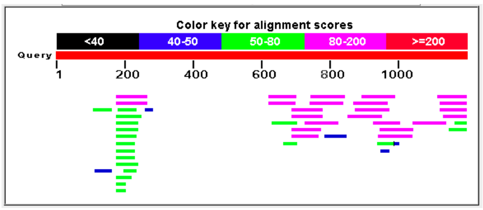

DRD4

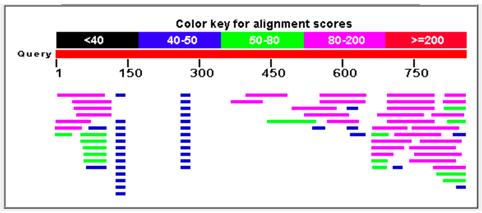

15. Red Throated Loon

Present: DRD1A, DRD1B, DRD1C, DRD1E, DRD2, DRD3
Unidentified: DRD4
Absent: N/A

15.1 Microsynteny Analysis

DRD4

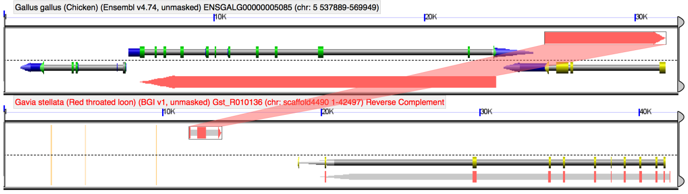

Regenerate Analysis: https://genomevolution.org/r/ffvn

15.2 Raw Reads BLAST Results

DRD1A

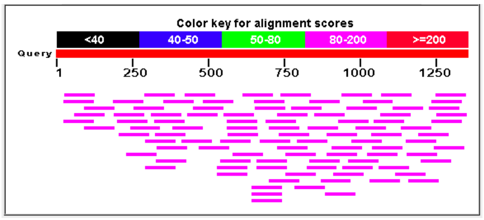

DRD4

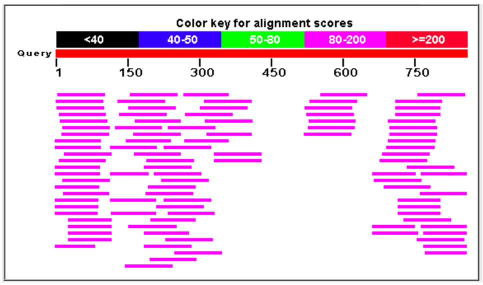

16. White Tailed Tropicbird

Present: DRD1A, DRD1B, DRD1C, DRD1E, DRD2, DRD3
Unidentified: DRD4
Absent: N/A

16.1 Microsynteny Analysis

DRD4

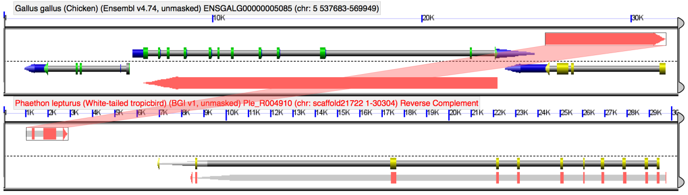

Regenerate Analysis: https://genomevolution.org/r/ffvu

16.2 Raw Reads BLAST Results

DRD1A

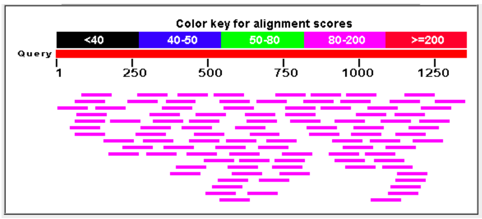

DRD4

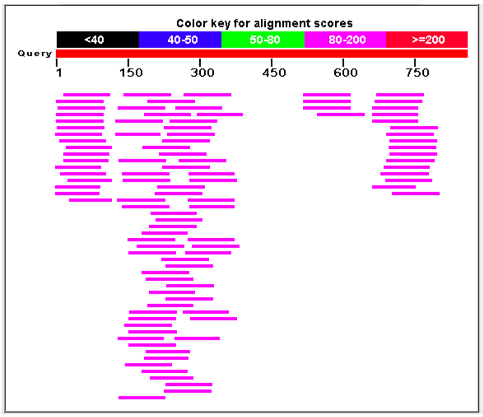

17. Sunbittern

Present: DRD1A, DRD1B, DRD1C, DRD1E, DRD2, DRD3
Unidentified: DRD4
Absent: N/A

17.1 Microsynteny Analysis

DRD4

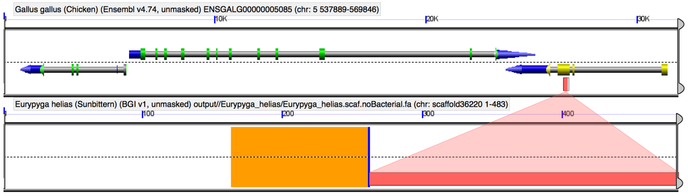

Regenerate Analysis: https://genomevolution.org/r/fjpv

17.2 Raw Reads BLAST Results

DRD1A

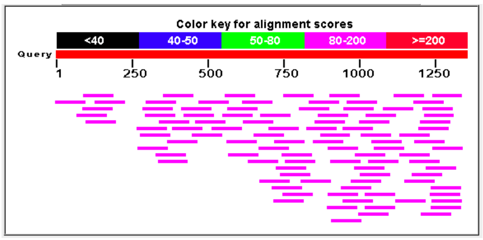

DRD4

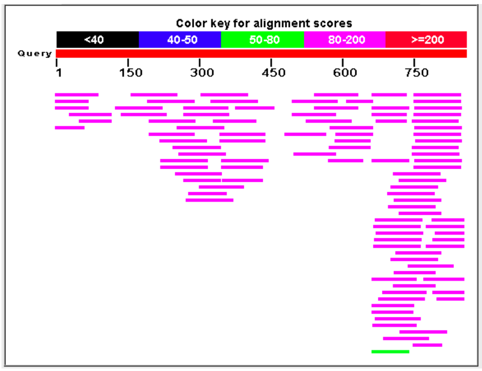

18. Killdeer

Present: DRD1A, DRD1B, DRD1C, DRD1E, DRD2, DRD3
Unidentified: DRD4
Absent: N/A

18.1 Microsynteny Analysis

DRD4

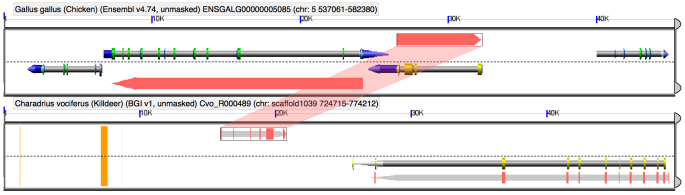

Regenerate Analysis: https://genomevolution.org/r/ffwe

18.2 Raw Reads BLAST Results

DRD1A

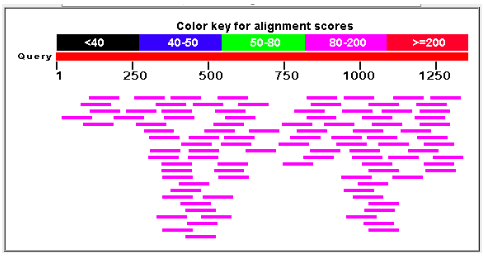

DRD4

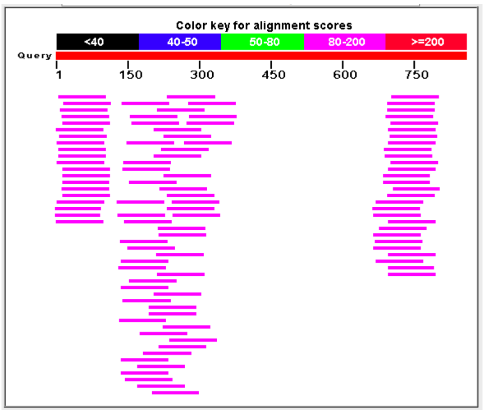

19. Hoatzin

Present: DRD1A, DRD1B, DRD1C, DRD1E, DRD2, DRD3
Unidentified: DRD4
Absent: N/A

19.1 Microsynteny Analysis

DRD4

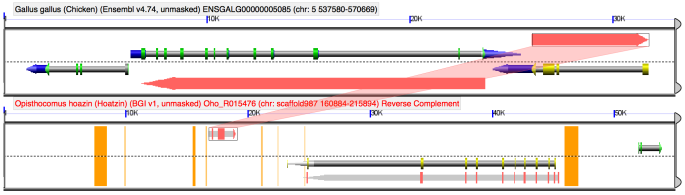

Regenerate Analysis: https://genomevolution.org/r/fg48

19.2 Raw Reads BLAST Results

DRD1A

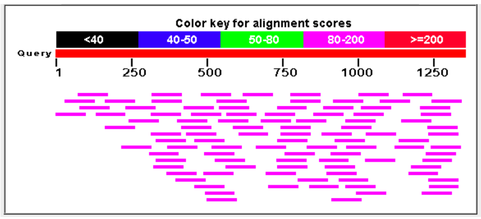

DRD4

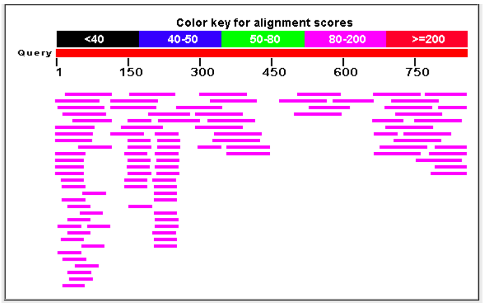

20. Anna's Hummingbird

Present: DRD1A, DRD1B, DRD2, DRD3
Unidentified: DRD1E, DRD4
Absent: DRD1C

20.1 Microsynteny Analysis

DRD1C

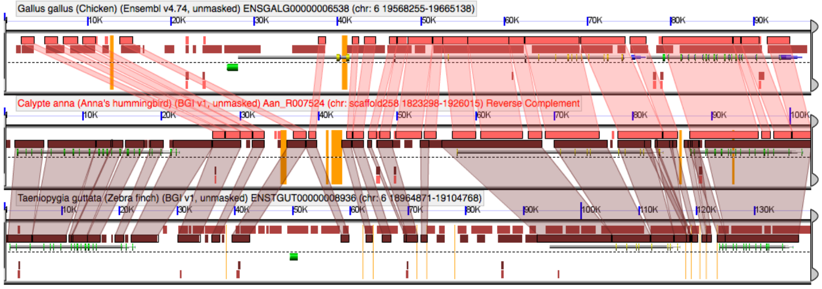

Regenerate Analysis: https://genomevolution.org/r/fgr3

DRD1E

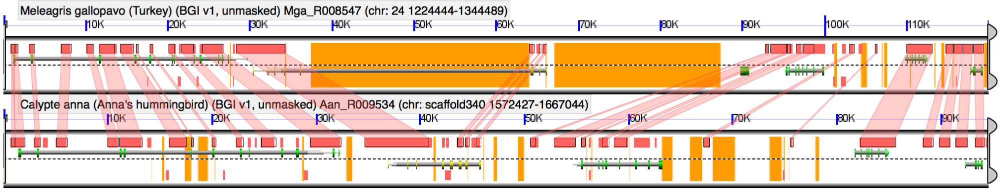

Regenerate Analysis: https://genomevolution.org/r/fizf

DRD4

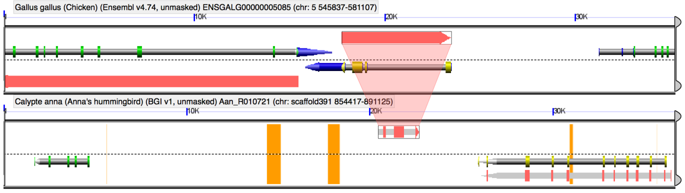

Regenerate Analysis: https://genomevolution.org/r/fg4h

20.2 Raw Reads BLAST Results

DRD1A

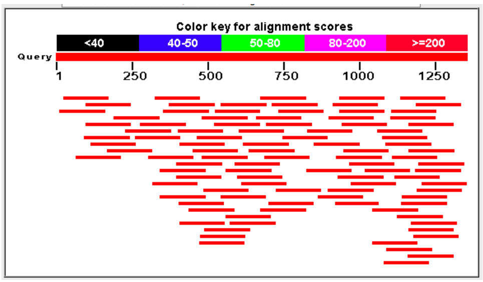

DRD1C

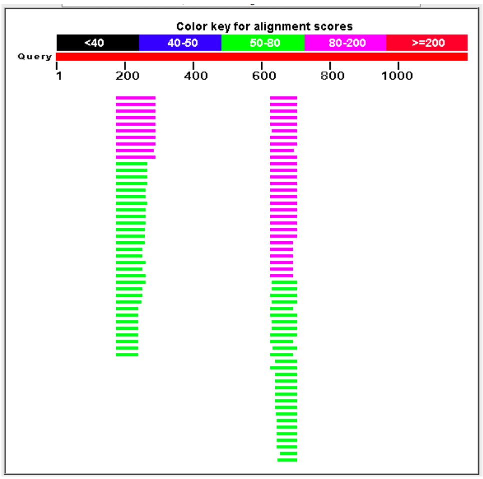

DRD1E

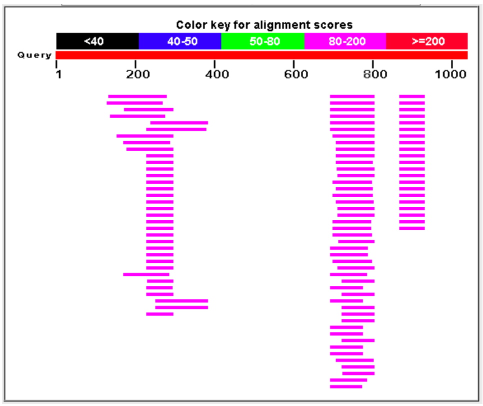

DRD4

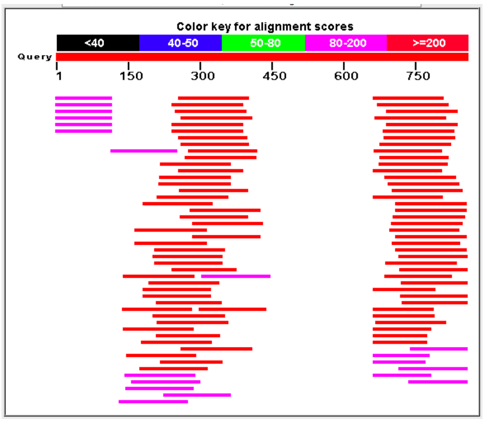

21. Chimney Swift

Present: DRD1A, DRD1B, DRD1C, DRD1E, DRD2, DRD3
Unidentified: DRD4
Absent: N/A

21.1 Microsynteny Analysis

DRD4

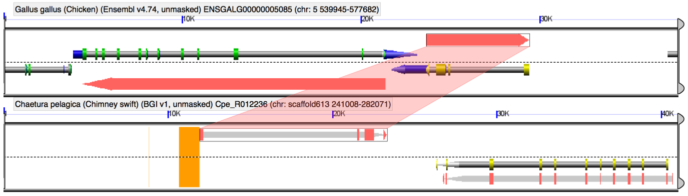

Regenerate Analysis: https://genomevolution.org/r/fg58

21.2 Raw Reads BLAST Results

DRD1A

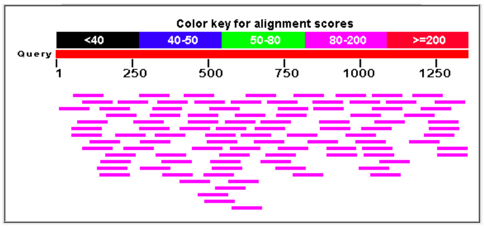

DRD4

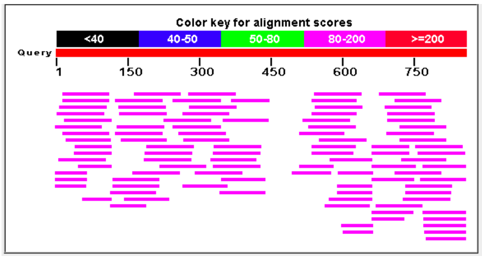

22. Macqueen Bustard

Present: DRD1A, DRD1B, DRD1C, DRD1E, DRD2, DRD3
Unidentified: DRD4
Absent: N/A

22.1 Microsynteny Analysis

DRD4

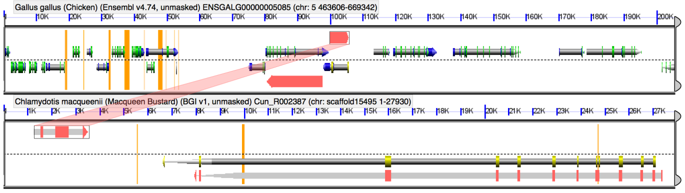

Regenerate Analysis: https://genomevolution.org/r/fg5t

22.2 Raw Reads BLAST Results

DRD1A

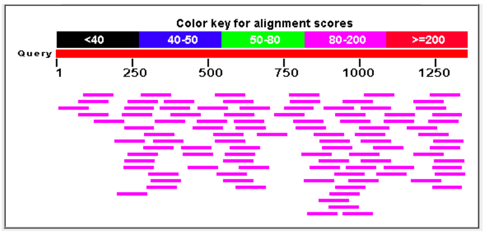

DRD4

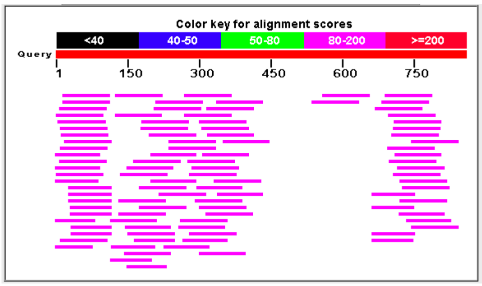

23. Angola Turaco

Present: DRD1A, DRD1B, DRD1C, DRD1E, DRD2, DRD3
Unidentified: DRD4
Absent: N/A

23.1 Microsynteny Analysis

DRD4

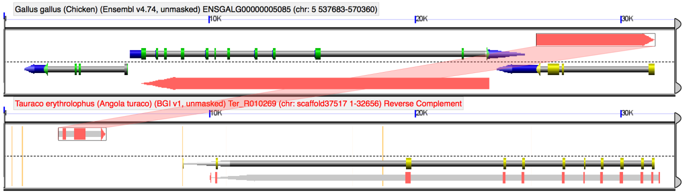

Regenerate Analysis: https://genomevolution.org/r/fg67

23.2 Raw Reads BLAST Results

DRD1A

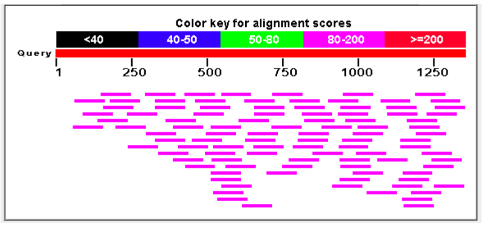

DRD4

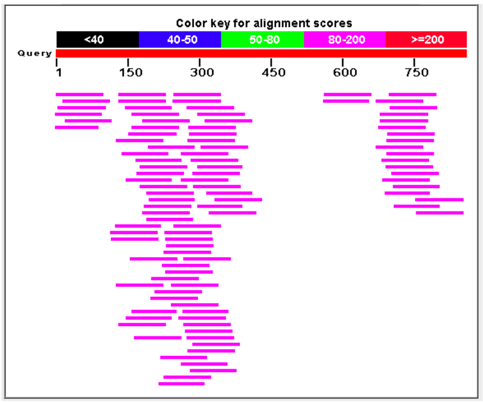

24. Brown Mesite

Present: DRD1A, DRD1B, DRD1C, DRD1E, DRD2, DRD3
Unidentified: DRD4
Absent: N/A

24.1 Microsynteny Analysis

DRD4

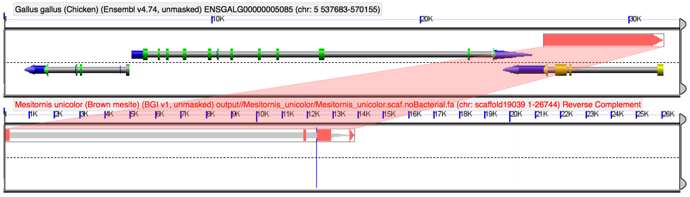

Regenerate Analysis: https://genomevolution.org/r/fg6l

24.2 Raw Reads BLAST Results

DRD1A

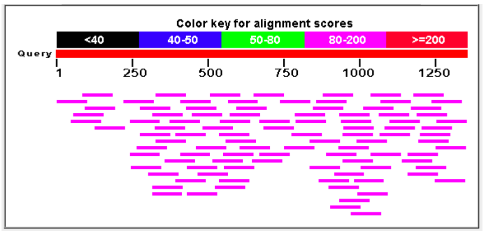

DRD4

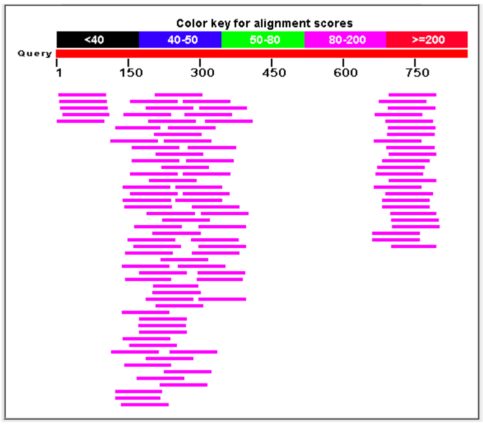

25. Yellow Throated Sandgrouse

Present: DRD1A, DRD1B, DRD1C, DRD1E, DRD2, DRD3
Unidentified: DRD4
Absent: N/A

25.1 Microsynteny Analysis

DRD4

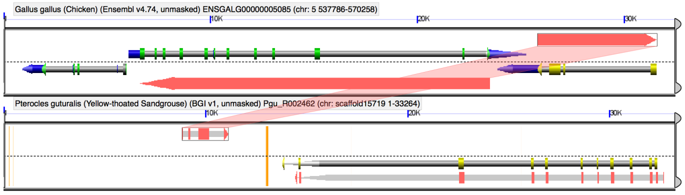

Regenerate Analysis: https://genomevolution.org/r/fg6u

25.2 Raw Reads BLAST Results

DRD1A

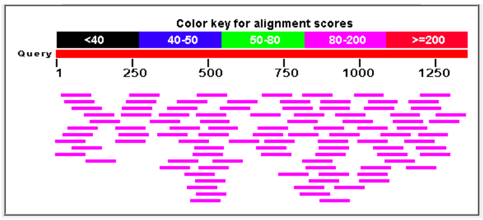

DRD4

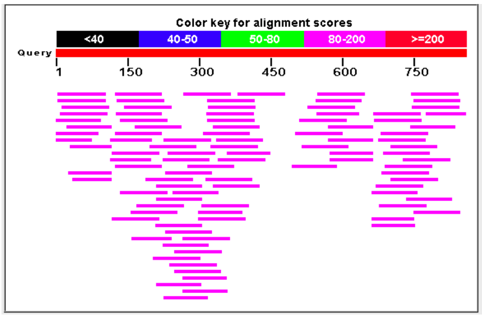

26. Domestic Pigeon

Present: DRD1A, DRD1B, DRD1C, DRD1E, DRD2, DRD3
Unidentified: DRD4
Absent: N/A

26.1 Microsynteny Analysis

DRD4

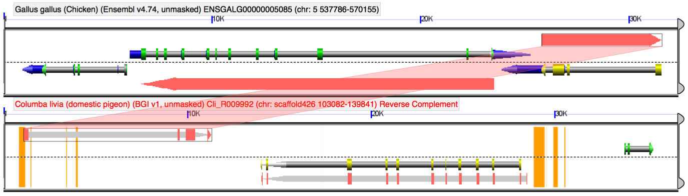

Regenerate Analysis: https://genomevolution.org/r/fg7d

26.2 Raw Reads BLAST Results

DRD1A

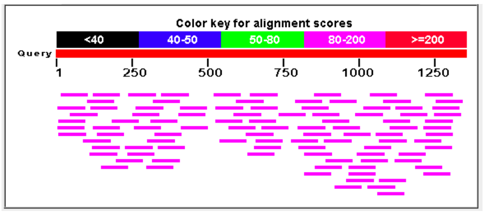

DRD4

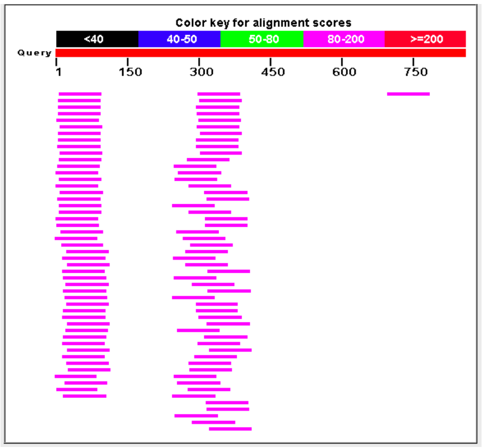

27. Great Crested Grebe

Present: DRD1A, DRD1B, DRD1C, DRD1E, DRD2, DRD3
Unidentified: DRD4
Absent: N/A

27.1 Microsynteny Analysis

DRD4

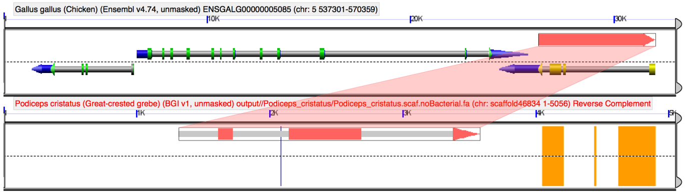

Regenerate Analysis: https://genomevolution.org/r/fg7t

27.2 Raw Reads BLAST Results

DRD1A

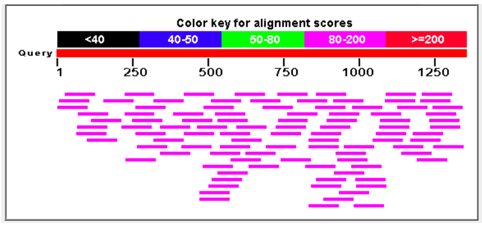

DRD4

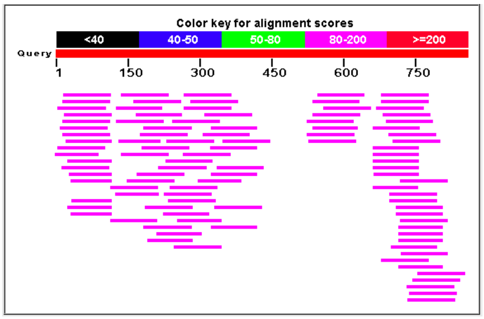

28. Chicken

Present: DRD1A, DRD1B, DRD1C, DRD2, DRD3, DRD4
Unidentified: DRD1E
Absent: N/A

28.1 Microsynteny Analysis

DRD1E

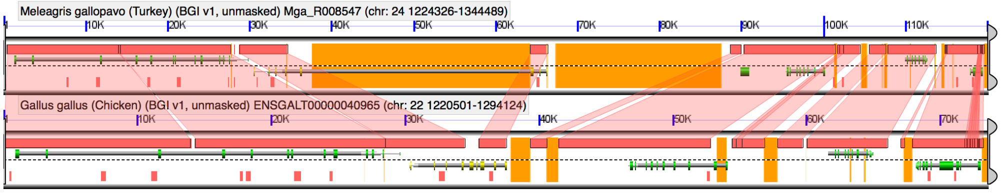

Regenerate Analysis: https://genomevolution.org/r/fj0j

28.2 Raw Reads BLAST Results

DRD1A

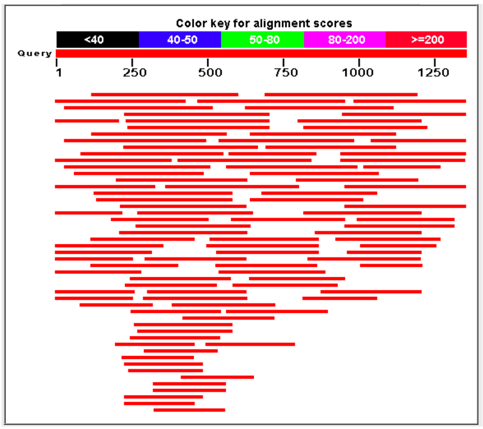

DRD1E

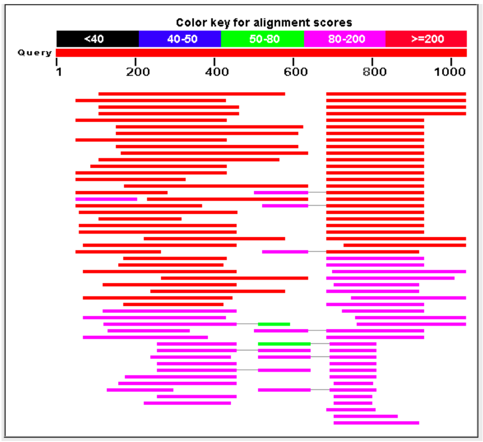

29. Turkey

Present: DRD1A, DRD1B, DRD1C, DRD1E, DRD2, DRD3
Unidentified: DRD4
Absent: N/A

29.1 Microsynteny Analysis

DRD4

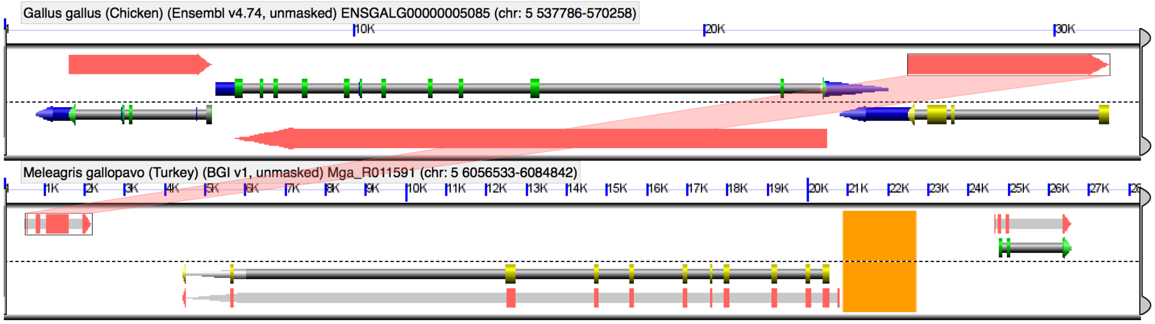

Regenerate Analysis: https://genomevolution.org/r/fg81

29.2 Raw Reads BLAST Results

Raw Sequencing Reads Unavailable

30. Peking Duck

Present: DRD1A, DRD1B, DRD1C, DRD1E, DRD2, DRD3
Unidentified: DRD4
Absent: N/A

30.1 Microsynteny Analysis

DRD4

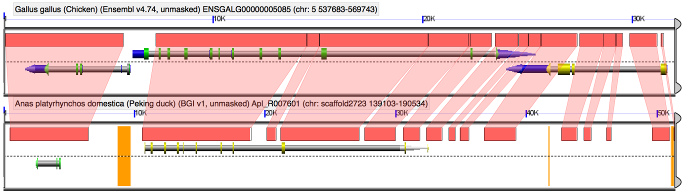

Regenerate Analysis: https://genomevolution.org/r/fg8i

30.2 Raw Reads BLAST Results

DRD1A

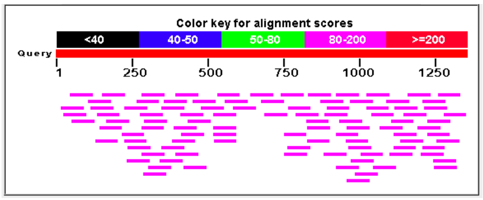

DRD4

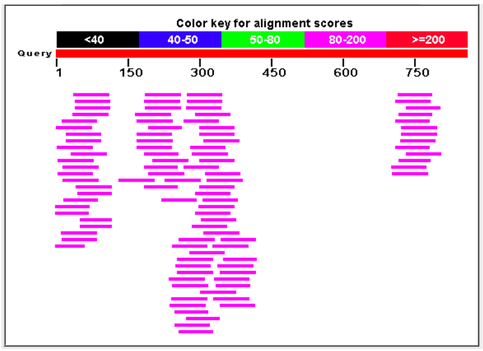

31. White Throated Tinamou

Present: DRD1A, DRD1B, DRD1C, DRD2, DRD3
Unidentified: DRD1E, DRD4
Absent: N/A

31.1 Microsynteny Analysis

DRD1E

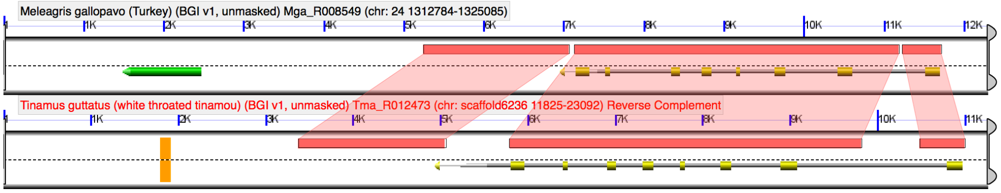

Regenerate Analysis: https://genomevolution.org/r/fj1m

DRD4

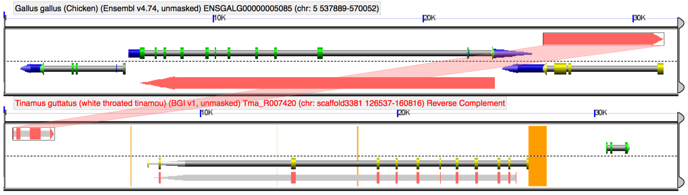

Regenerate Analysis: https://genomevolution.org/r/fg8m

31.2 Raw Reads BLAST Results

DRD1A

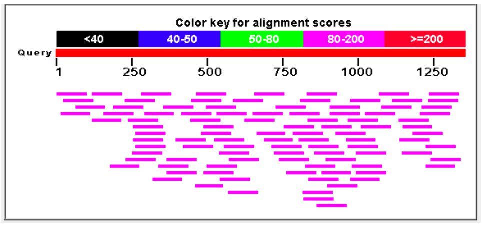

DRD1E

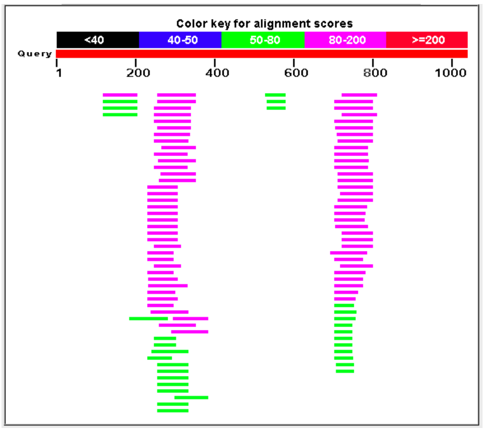

DRD4

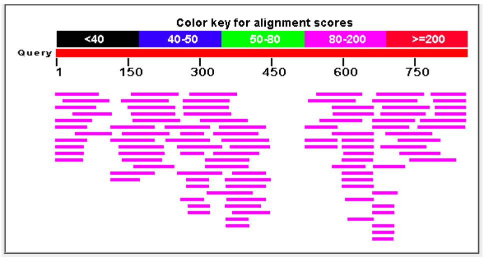
Color key for alignment scores
<40
40-50
50-80
80-200
>=200
Query
1
150
300
450
600
750

32. Ostrich

Present: DRD1A, DRD1B, DRD1C, DRD1E, DRD2, DRD3
Unidentified: DRD4
Absent: N/A

32.1 Microsynteny Analysis

DRD4

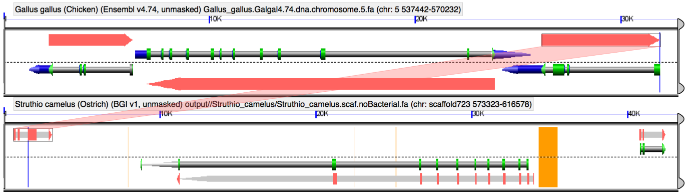

Regnerate Analysis: https://genomevolution.org/r/fg8s

32.2 Raw Reads BLAST Results

DRD1A

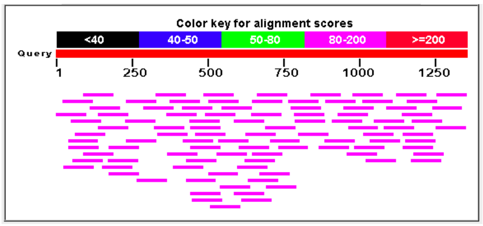

DRD4

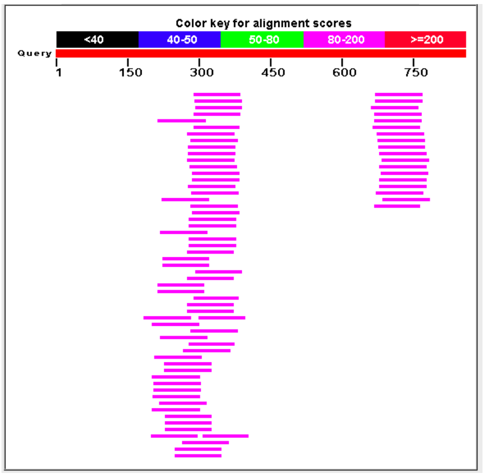

Supplement: Supplementary file 1 [file DataSheet1.PDF]
