# Supplementary material for: Identification of dopamine receptors across the extant avian family tree and analysis with other clades uncovers a polyploid expansion among vertebrates
Source: Front Neurosci. 2015 Oct 7;9:361. doi: 10.3389/fnins.2015.00361 (PMC4595791; doi:10.3389/fnins.2015.00361)
Supplement: Supplementary file 2 [file DataSheet2.PDF]

Supplementary File 2

Multiple Sequence Alignment Assessment

To assess validity of the automated multiple sequence alignments produced by MUSCLE 3.8.31 (Edgar, 2004) (Supplementary files 5, 6), a subset of the DA receptor alignment was selected for manual optimization and comparison. The subset of sequences is composed of all identified DRD1C receptors and the Ciona D1-like receptor as an outgroup. DRD1C receptors were selected because they demonstrated the largest number of areas that would benefit from manual optimization. Based on the findings of this assessment that showed little, if any, improvement in the resulting phylogenetic trees, automatically optimized alignments were used for all analyses. In addition, automated alignments increase the reproducibility of our analyses. It is worth noting that the quality of the alignments used may have minor impacts on species-level relationships and branch support values (describe below), but does not impact the overall findings of the study regarding the polyploidy origins of DRD1A/DRD1B and DRD2/DRD3 receptors, and the subsequent receptor losses that have occurred in some lineages of birds.

Figure S1. DRD1C automatically (A) and manually optimized (B) multiple sequence alignments.
(A) Sequences were automatically aligned using an iPlant implementation of MUSCLE 3.8.31.
(B) The alignment from (A) was manually optimized using Jalview (Waterhouse et al., 2009) to correct minor errors in the alignment that may influence phylogenetic reconstruction. Red boxes highlight areas manually modified to optimize the alignment. These alignments were used to generate Figures S2 and S3.

Figure S2. DRD1C phylogenetic relationships with bootstrap support values built from automatically optimized (A) and manually optimized (B) sequence alignments.
Phylogenetic relationships between DRD1C receptors marked with bootstrap support values. Red boxes indicate nodes with rearrangements, but with very low support values in both trees. Green box indicates the single node with changes in taxa relationships and a relatively large change (but still low value) in bootstrap support, which was higher under the automatically optimized alignment. As highlighted through the boxes, minor rearrangements and support value are seen through manual optimization, but do not improve the accuracy of the observed relationships with the currently accepted species-level relationships (Jarvis, 2014). Phylogenetic trees were generated with RAxML using the rapid bootstrap and best-scoring ML tree single run algorithm. Calculations were performed using the PROTGAMMA model and WAG substitution matrix, and 100 bootstrap replicates were performed to provide support values. Tree is rooted on the Ciona D1-like receptor.

Figure S3. DRD1C Phylogenetic relationships with branch lengths built from automatically optimized (A) and manually optimized (B) sequence alignments.
Phylogenetic relationships between DRD1C receptors with branch lengths indicated. Minor variation in branch length is observed, but manual optimization does not improve the quality of the phylogeny (Figure S2) suggesting that the overall impact of manually optimizing the alignments is negligible. Trees were generated with protocol dictated in Figure Legend S2.

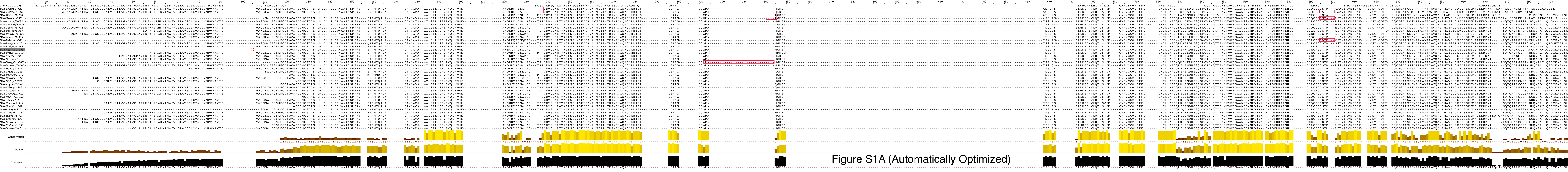

Figure S1A (Automatically Optimized)

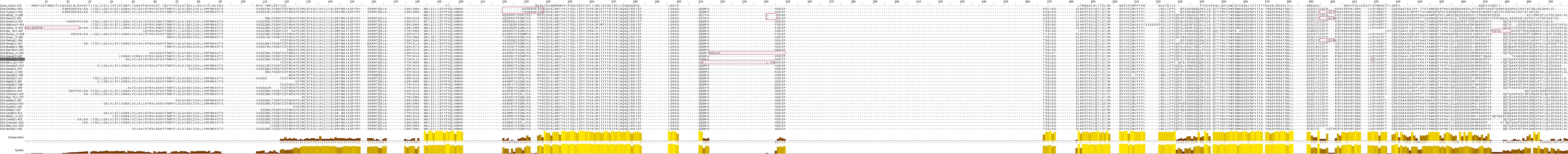

Figure S1B (Manually Optimized)

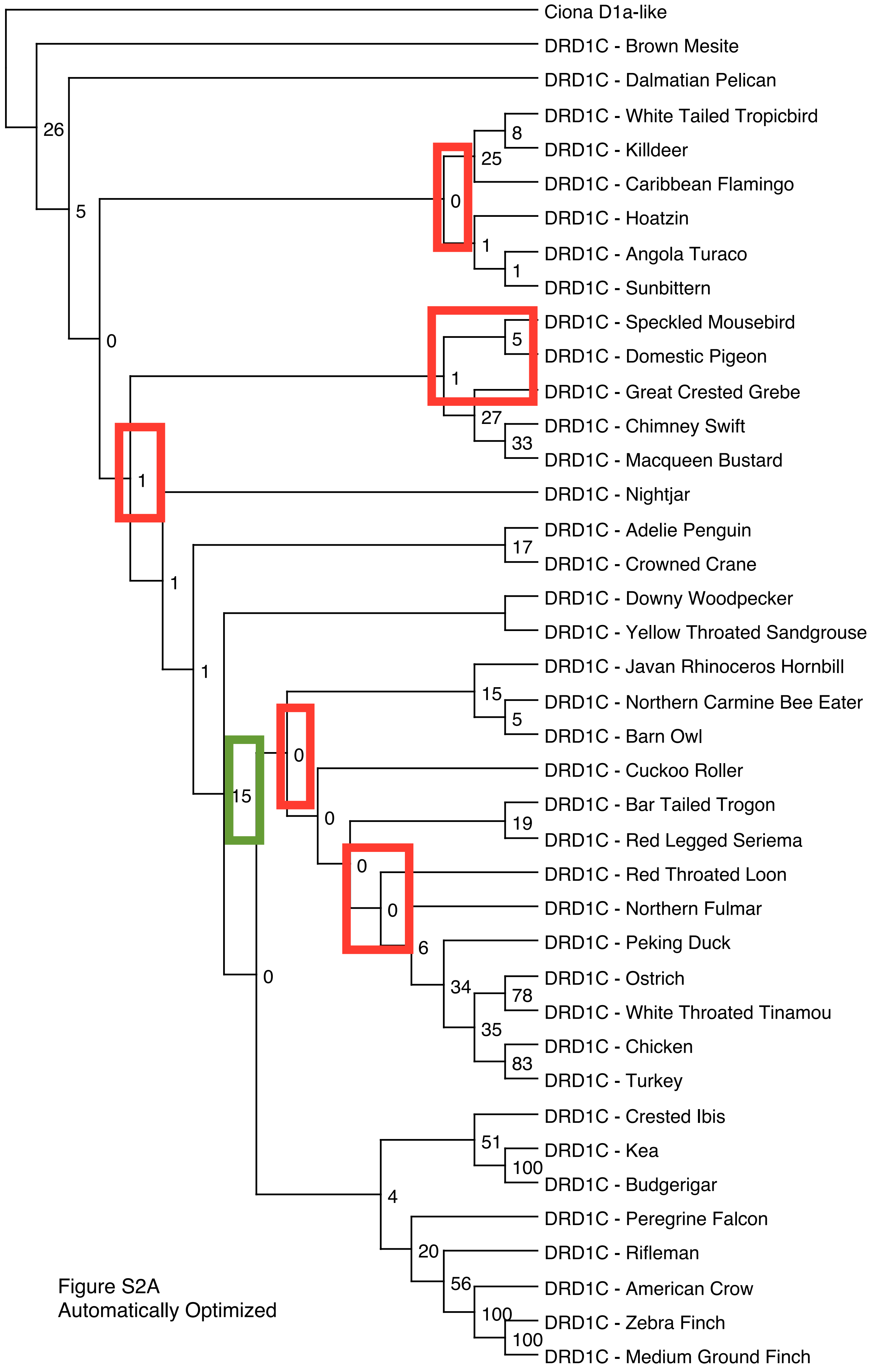

Figure S2A
Automatically Optimized

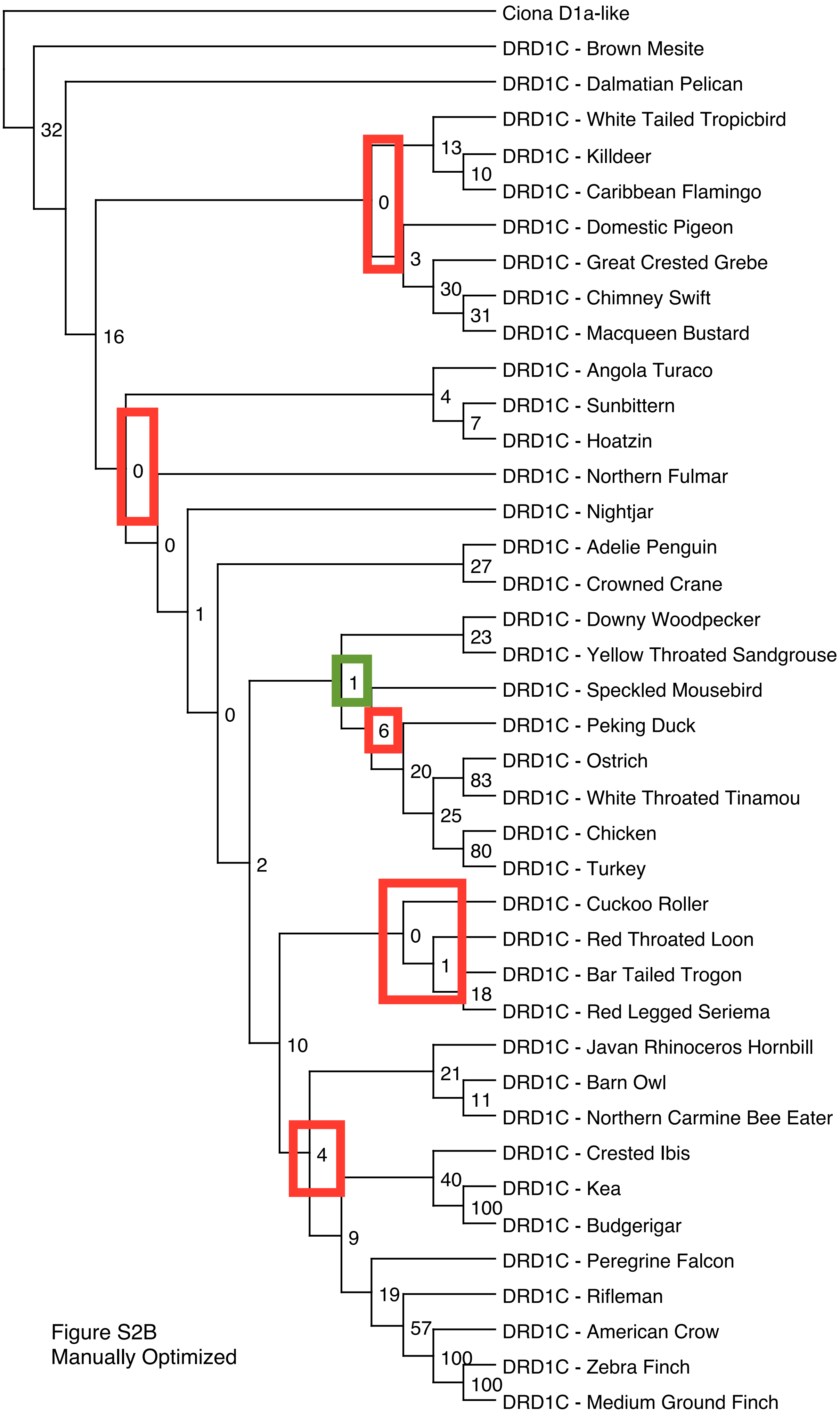

Figure S2B
Manually Optimized

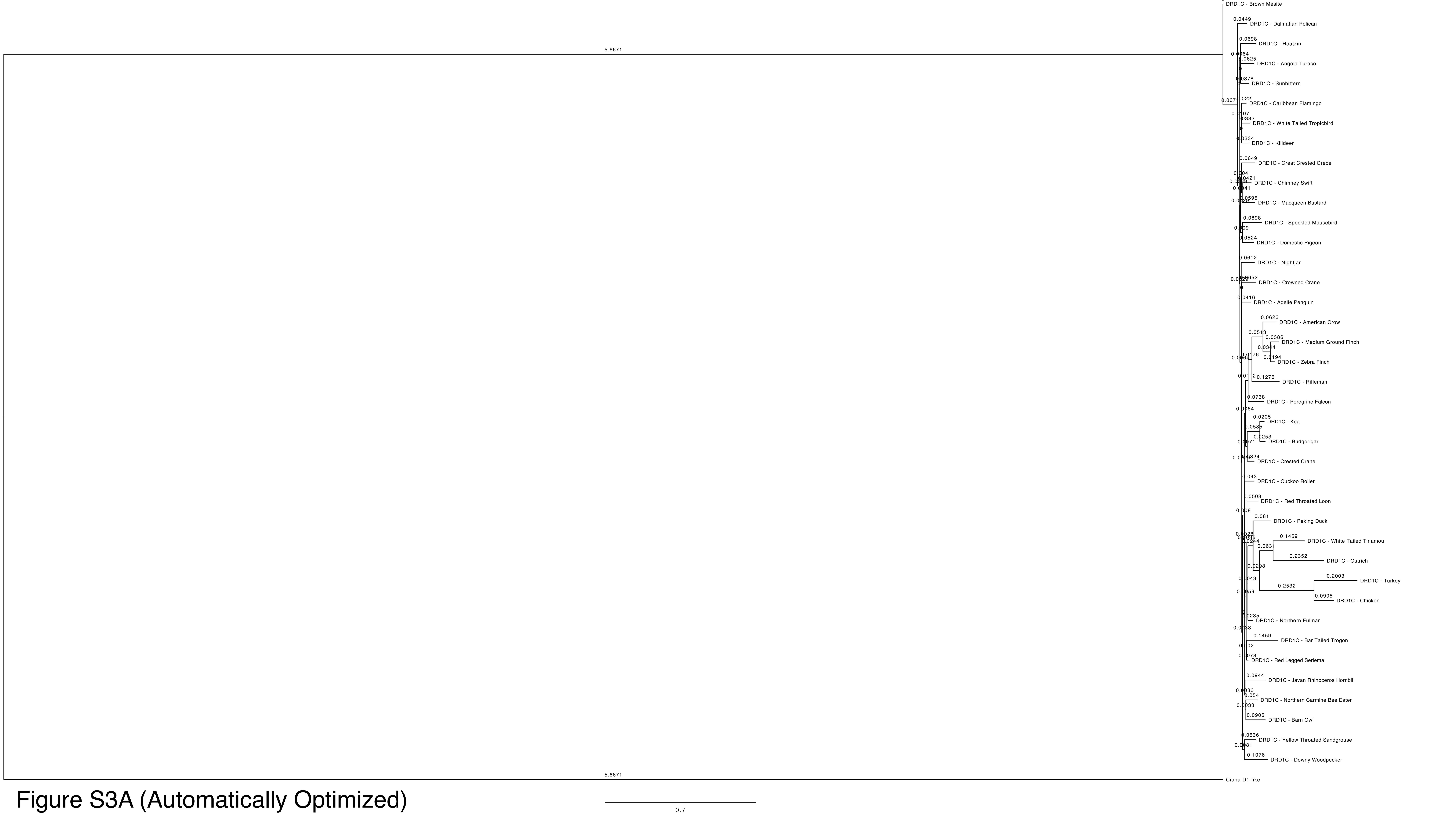

Figure S3A (Automatically Optimized)

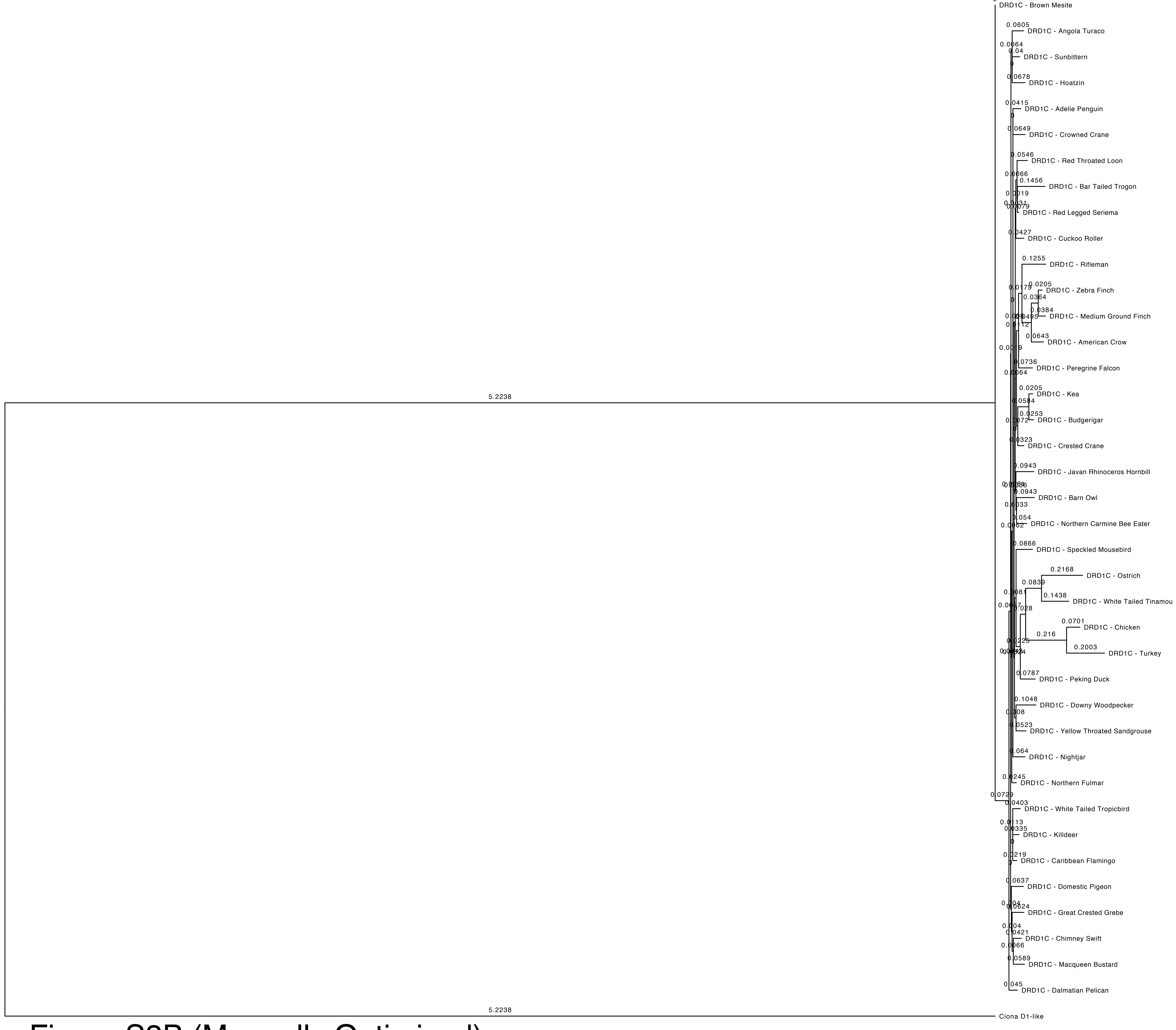

Figure S3B (Manually Optimized)
