# Supplementary material for: Identification of dopamine receptors across the extant avian family tree and analysis with other clades uncovers a polyploid expansion among vertebrates
Source: Front Neurosci. 2015 Oct 7;9:361. doi: 10.3389/fnins.2015.00361 (PMC4595791; doi:10.3389/fnins.2015.00361)

Supplementary File 3

Whole Genome Duplications and Dopamine Receptor Evolution in Vertebrate/Invertebrate Lineages

Identification of Fugu D1 genes for construction of an evolutionary phylogeny identified two putative genes each for gene families DRD1A, DRD1B and DRD1C. Further investigation (both in literature and our analyses) suggested that these duplicates might have originated from an additional round of whole genome duplication that occurred in the teleost fish lineage (Panopoulou and Poustka, 2005). Here, we document this duplication and provide microsynteny evidence for the fugu DRD1A/DRD1B genes used in construction of Figure 7.

Figure S4. Phylogeny of select species with WGD events indicated.
Phylogenetic relationships between drosophila, ciona, lamprey, fugu, alligator, turkey chicken, mouse, and human. Whole genome duplications are indicated by red stars (Panopoulou and Poustka, 2005). In addition to the two rounds of whole genome duplication in the vertebrate lineage, an additional round can be seen in the fugu (teleost fish) lineage.

Figure S5. Phylogeny of DA receptor genes.
Phylogenetic relationship of D1 and D2 dopamine receptors for two invertebrate (Drosophila and Ciona D1-like) and seven vertebrates (Lamprey, Fugu, Alligator, Turkey, Chicken, Mouse and Human) species based on protein coding sequences. Bootstrap support values are shown at nodes; branch lengths (substitutions/changes per site) are shown with branches. Phylogeny can be regenerated with respective multi-FASTA file (Supplementary File 5) or multiple sequence alignment (Supplementary File 7) using methods outlined in main text.

Figure S6. Whole genome comparison of fugu/chicken.
(A) Syntenic dotplot between chicken (x-axis) and fugu (y-axis) with an enforcement of a 2:1 syntenic relationship between fugu and chicken, respectively. Vertical and horizontal grey lines separate chromosomes; dots are syntenic gene pairs that a colored by Ks values. Red boxes highlight regions of where there is a two to one syntenic relationship of fugu and chicken genomic regions. (B) Histogram of log10 transformed Ks values for syntenic gene pairs identified in (A). Taken together, the evidence supports an additional round of whole genome duplication in the fugu lineage with respect to avian and mammal lineages. Analysis may be regenerated at https://genomevolution.org/r/hzjg.

Figure S7. Microsynteny confirmation of fugu DRD1A(1/2) (A) and DRD1B(1/2) (B) genes.
Microsynteny validation of BLAST identified fugu DRD1A1/DRD1A2 (A) and DRD1B1/DRD1B2 (B) genes. Detailed instructions on how to read microsynteny analyses are described in Figure 3 legend. For each comparison, the top and bottom panels are putative Fugu DA receptor genes, compared to their orthologous chicken gene in the center panel. A colinear order of genes surrounding the putative genes suggests that they are correctly identified and have originated from a common ancestor. Each analysis can be regenerated using the links in the figure.

Figure S4: Species-level phylogeny with WGD events indicated.

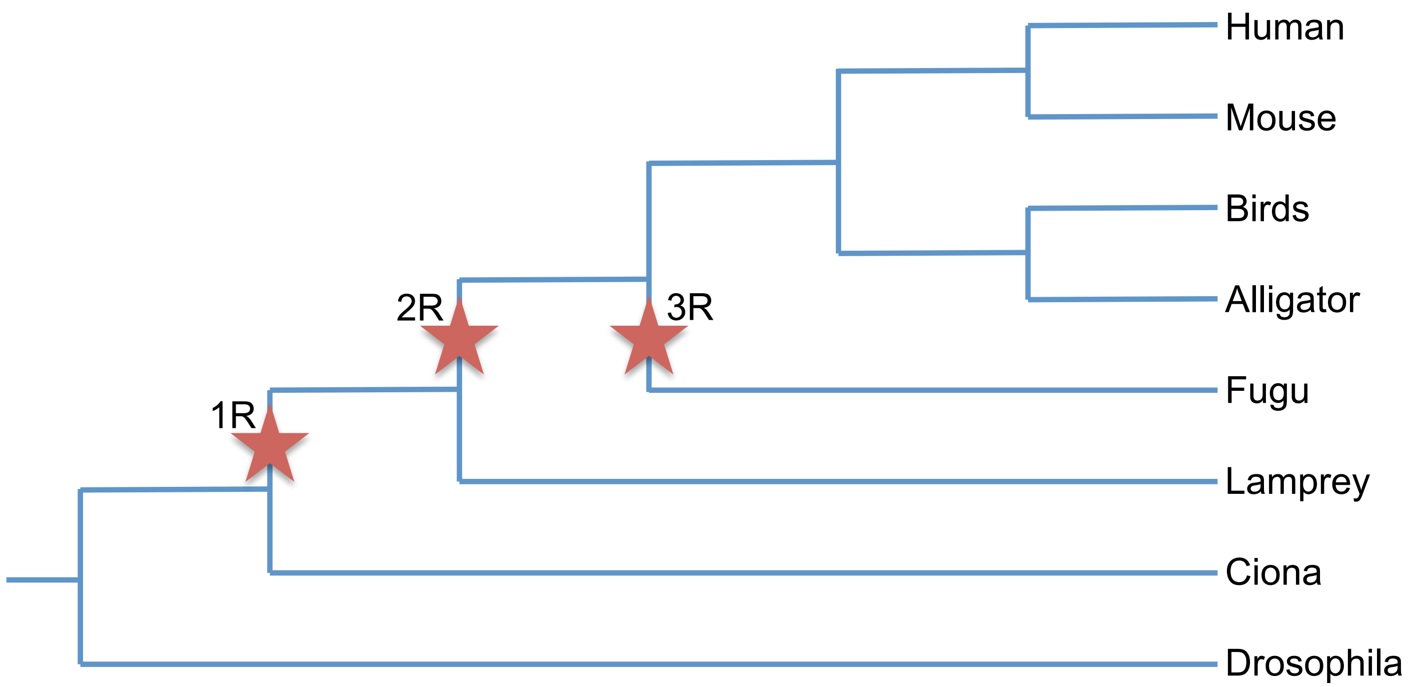

Figure S5: Phylogeny of DA receptor genes

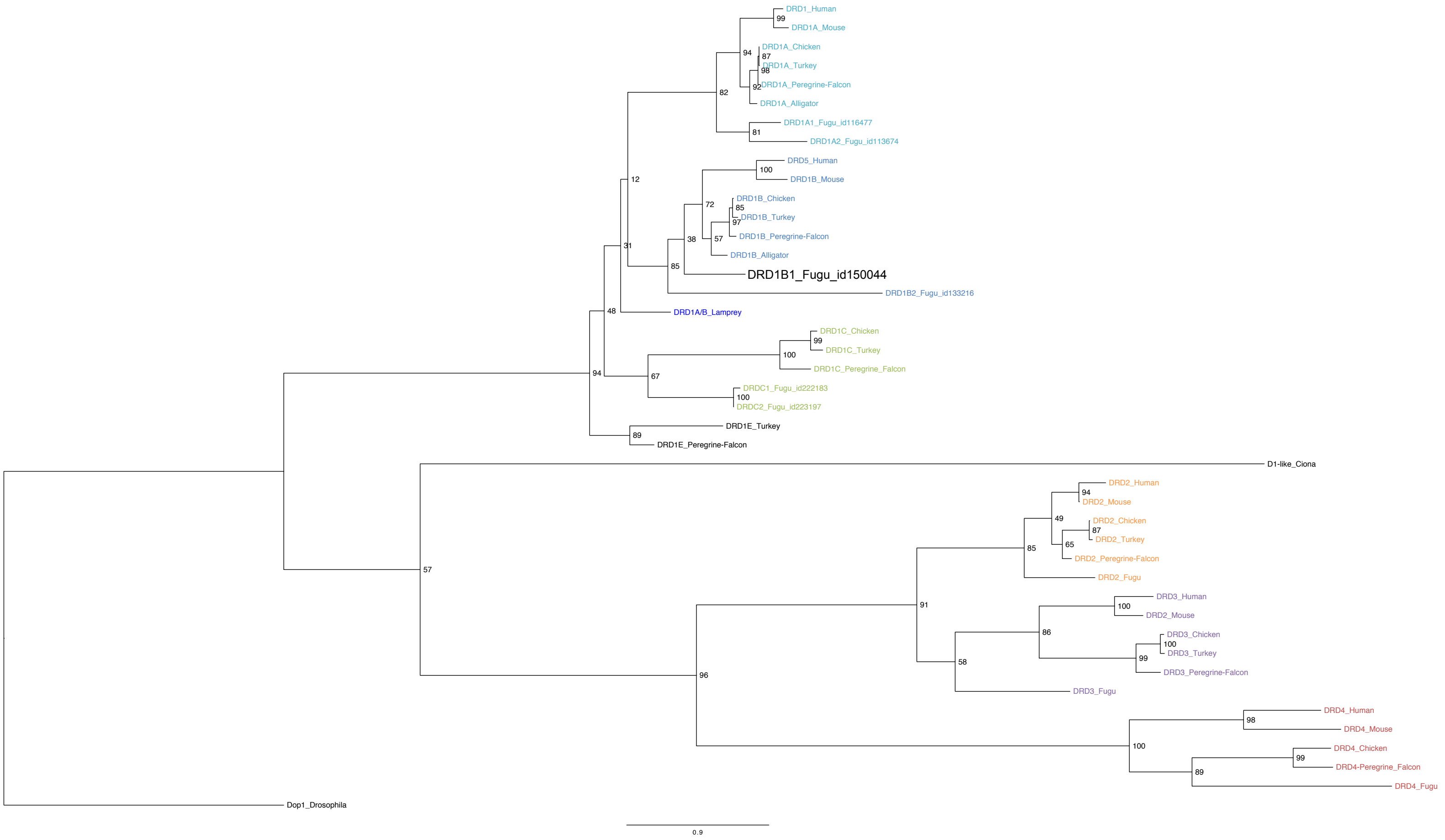

Figure S6: Whole genome comparison of Chicken/Fugu.

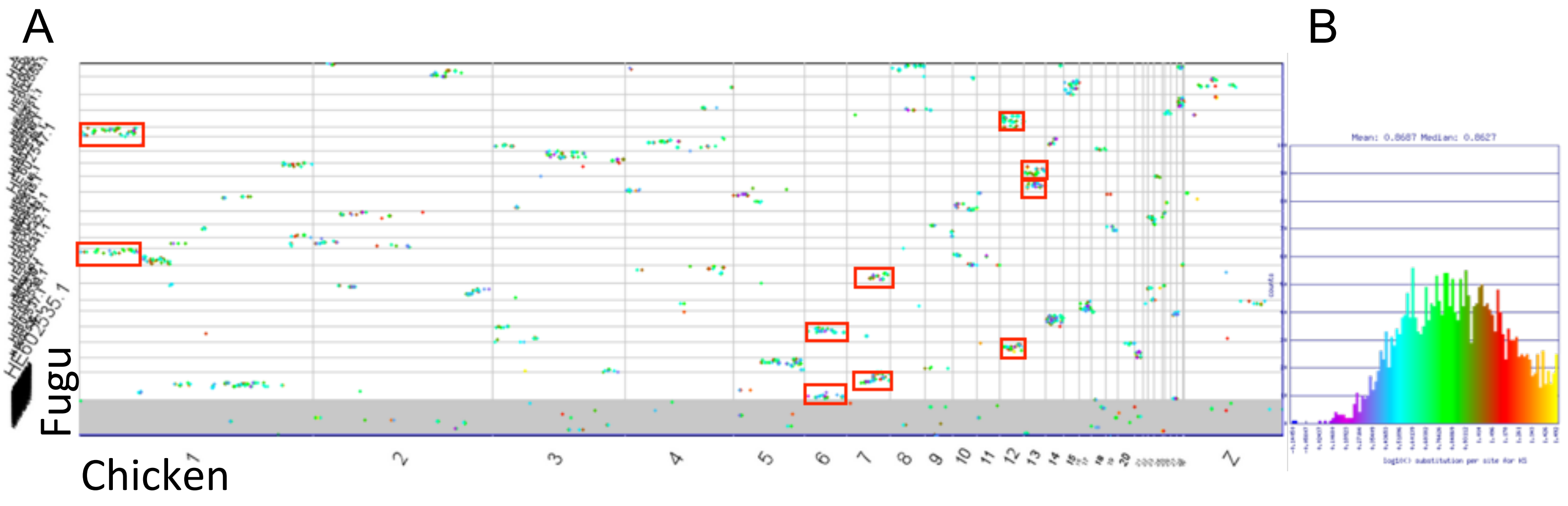

Figure S7: Microsynteny confirmation of Fugu DRD1A1/2 (A) and DRD1B1/2 (B).

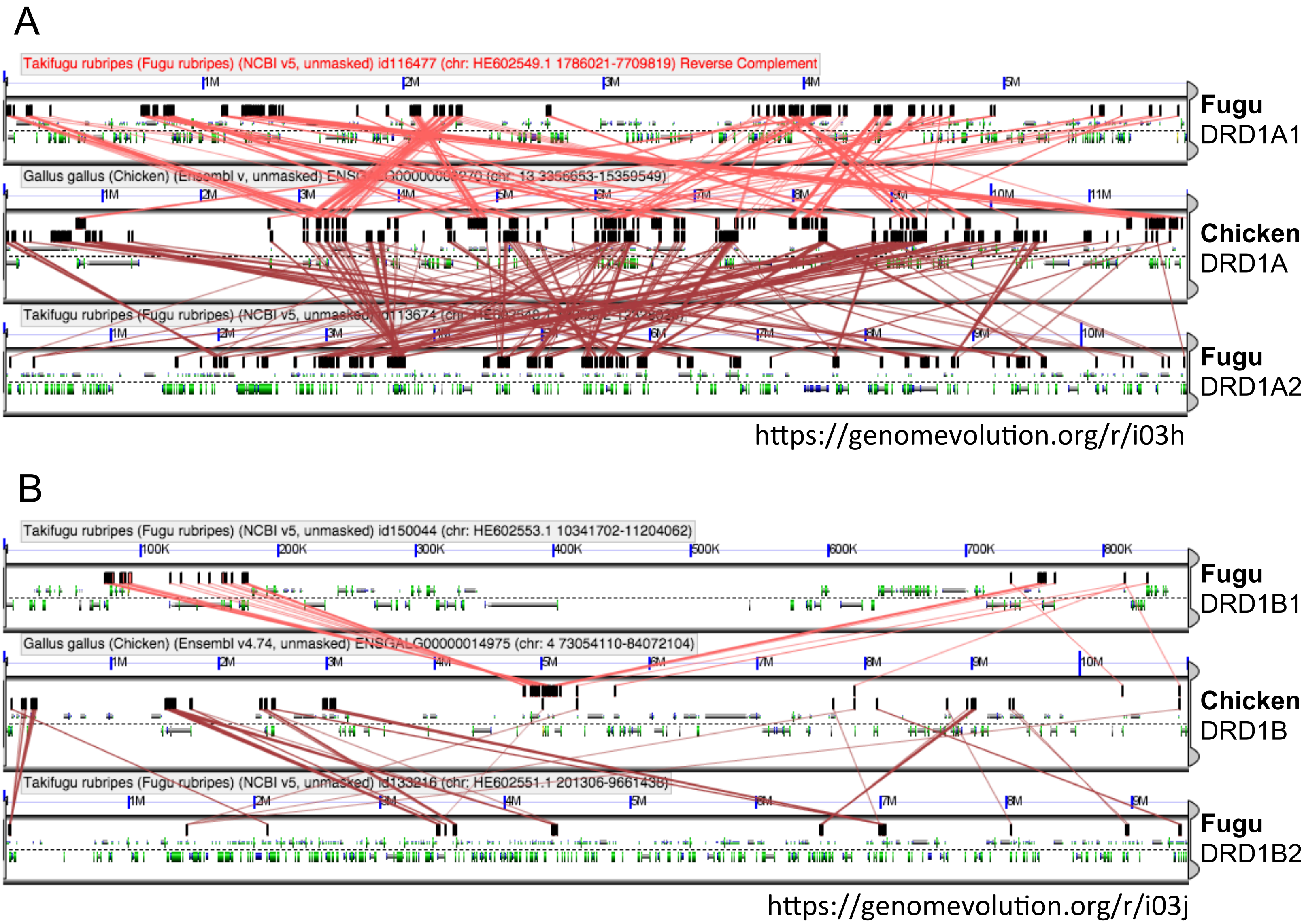

Supplement: Supplementary file 3 [file DataSheet3.PDF]
